# A study of RNA splicing and protein expression in the living human brain

**DOI:** 10.1371/journal.pone.0332651

**Published:** 2025-10-09

**Authors:** Brian H. Kopell, Deepak A. Kaji, Lora E. Liharska, Eric Vornholt, Anina Lund, Alice Hashemi, Ryan C. Thompson, Jessica S. Johnson, Nicole Bussola, Esther Cheng, You Jeong Park, Punit Shah, Weiping Ma, Richard Searfoss, Gregory M. Miller, Nischal Mahaveer Chand, Jack Humphrey, Lillian Wilkins, Kimia Ziafat, Hannah Silk, Lisa M. Linares, Brendan Sullivan, Claudia Feng, Vanessa Cohen, Prashant Kota, Emily Moya, Marysia-Kolbe Rieder, Girish N. Nadkarni, Michael S. Breen, Joseph Scarpa, Niven R. Narain, Pei Wang, Michael A. Kiebish, Eric E. Schadt, Noam D. Beckmann, Alexander W. Charney

**Affiliations:** 1 Icahn School of Medicine at Mount Sinai, New York, New York, United States of America; 2 BPGbio, Waltham, Massachusetts, United States of America; Universitatsklinikum Hamburg-Eppendorf, GERMANY

## Abstract

Due to the unavailability of living human brain tissue for molecular research, postmortem brain samples are currently the standard tissue source for molecular studies of the human brain. The Living Brain Project (LBP) was designed to test the assumption that the postmortem brain is an accurate molecular representation of in the living brain on multiple levels of molecular biology. Findings from previous LBP reports suggest that this assumption does not hold with respect to RNA transcript expression levels. Here, molecular differences between living and postmortem human prefrontal cortex tissues obtained for the LBP are corroborated through analyses of RNA splicing and protein expression data. Significant differences were observed with respect to (1) the expression of most primary RNA transcripts, mature RNA transcripts, and proteins, (2) the splicing of most primary RNA transcripts into mature RNA transcripts, and (3) the patterns of co-expression between RNA transcripts and proteins. Taken together, this report corroborates the presence of widespread molecular differences between living and postmortem human brain tissues. These observations should be considered when designing and interpreting studies of human brain biology.

## Introduction

The central dogma of molecular biology states that from specialized sequences of DNA (“genes”), raw forms of messenger RNA (“primary RNA transcripts”) are transcribed, which are cut and pasted (“spliced”) into processed forms (“mature RNA transcripts”) that are then translated into proteins [1]. In this report, the coordinated processes involving many thousands of RNA transcripts and proteins that ultimately give rise to tissue function are referred to as the molecular foundations of tissue function [2]. Due to the unavailability of living human brain tissue for molecular research, postmortem brain samples are the standard tissue source for studies of the molecular foundations of human brain function. Many such studies are intended to discover the molecular foundations of brain functions in living people (e.g., the molecular processes that are dysfunctional in the brain of a person with schizophrenia) so that subsequent steps can be taken towards improving human health (e.g., the development of a medication that fixes the dysfunctional molecular processes in the brain of a person with schizophrenia). These studies are conducted based on the implicit assumption that the postmortem brain is an accurate molecular representation of the living brain.

**Fig 1:**
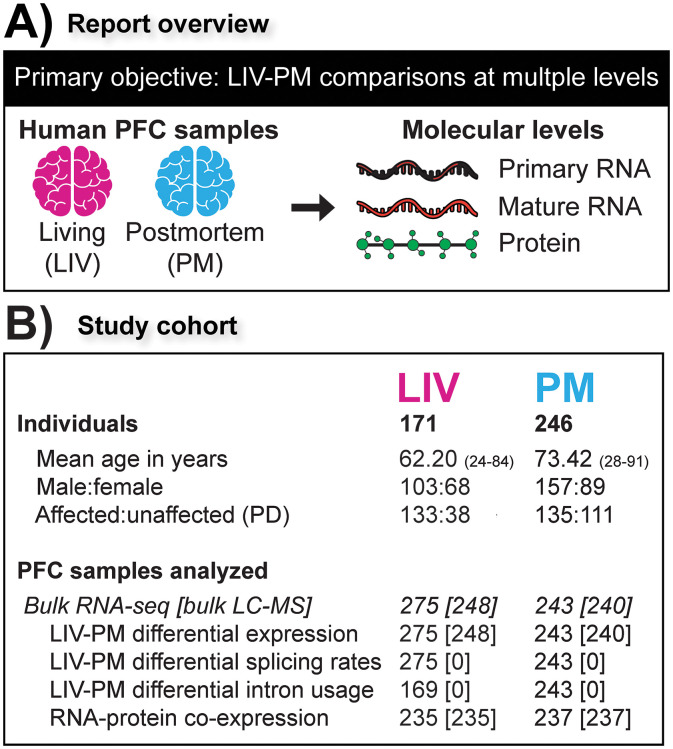
(A) Report overview. Schematic illustrating the study objective and how it was achieved. **(B)** Study cohort. Numbers refer to sample size (i.e., individuals or samples) except for age. Sample sizes are shown for most of the analyses of LBP data presented in the report. Sample sizes inside and outside of the square brackets indicate counts for bulk RNA-seq and bulk LC-MS analyses, respectively.

Historically, studies testing this assumption in humans have been small in scale, conducted prior to the advent of next-generation sequencing biotechnologies, and limited to comparisons of living and postmortem cohorts not matched for key clinical and technical variables [3–6]. On this historical backdrop, the Living Brain Project (LBP) was designed to rigorously compare living and postmortem human brain samples at multiple molecular levels by developing a safe and scalable procedure to acquire prefrontal cortex (PFC) tissue from living people for biomedical research purposes (Fig 1) [7–9]. In the flagship report on LBP samples, Liharska *et al.* compared RNA transcript expression between 275 PFC samples from living participants and 243 PFC samples from postmortem donors, identifying significant differences in expression levels for approximately 80% of the RNA transcripts examined and transcriptome-wide changes in co-expression networks [8]. Here, through analyses of new and previously reported data, the LBP comparison of living and postmortem human PFC samples is expanded to the levels of RNA splicing and protein expression.

## Results

### Living brain project cohort

As described in other LBP reports [7,8], a procedure was developed for the LBP to obtain PFC samples from living study participants for research purposes during neurosurgical procedures for deep brain stimulation (DBS), an elective treatment for neurological and mental illnesses [10]. A total of 288 PFC biopsies (“LIV samples”) from 171 living participants were studied for the current report, including unilateral biopsies from 54 participants (40 from the left hemisphere and 14 from the right hemisphere) and bilateral biopsies from 117 participants. For comparison to LIV samples, a cohort of postmortem PFC samples (“PM samples”, N = 246) was assembled from three brain banks. To the extent that it was possible, PM samples were matched to LIV samples for age, sex, and the size of tissue used for RNA and protein extraction. The majority of samples were obtained from individuals with Parkinson’s disease (PD; Fig 1B), the most common indication for DBS.

All of the LIV samples and PM samples studied in the current report have been studied in a previous LBP report by Liharska *et al*. [8], though the current report introduces new data linked to these samples. The analyses presented in the main text of the current report center around two LBP datasets (Fig 1B): (1) bulk RNA-seq data from 518 PFC samples (275 LIV samples and 243 PM samples) introduced in the LBP report by Liharska *et al.*[8]; (2) bulk liquid chromatography-mass spectrometry (bulk LC-MS; i.e., the capture and quantification of pooled protein from the cells of a sample) data from 488 PFC samples (248 LIV samples and 240 PM samples; this dataset is introduced in the current report). Analyses presented in the S1 File of the current report utilize two additional LBP datasets: (1) immunohistochemistry data from 458 PFC samples, a subset of which was introduced in Liharska *et al.*[8]; (2) clinical data introduced in Liharska *et al*. [8]. Each of the LBP datasets analyzed is more fully described in Liharska *et al.*[8] (when applicable), the respective section of the current report that first describes results of analyses of the dataset, and/or in the methods section of the current report. The details regarding the procedures used for data quality control and statistical modeling these data are fully described in the methods section of the current report (S1–4 Figs).

### Primary RNA, mature RNA, and protein expression levels differ between LIV and PM samples

When the expression levels of molecular features (i.e., RNA transcripts, proteins) have been characterized for a given set of samples, differential expression (DE) analysis can be performed to characterize the association between the expression level of every feature and a trait of interest using a regression model. For a given feature, the regression model beta (by convention, the “logFC” value) for the trait of interest captures both the magnitude and direction of the feature-trait association. In this report, the set of feature-trait logFCs for all features tested in a DE analysis is referred to as the “DE signature” of the trait, and features with statistically significant associations with the trait are referred to as “differentially expressed features” (DEFs). The trait of primary interest for the DE analyses in this report is “LIV-PM status” (i.e., whether a PFC sample is from a living participant or a postmortem donor).

**Fig 2:**
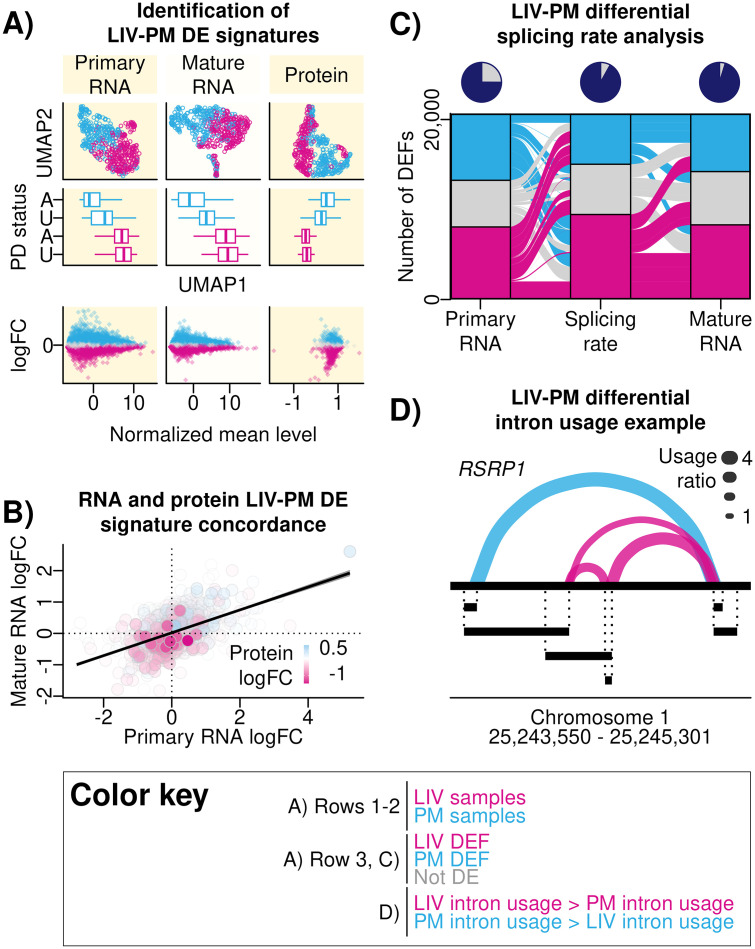
(A) Identification of LIV-PM DE signatures. A matrix of nine plots is presented that altogether summarize the differences in RNA and protein expression levels between LIV samples and PM samples. Each row of the matrix is a different analysis, and each column is a different data type (primary RNA, mature RNA, protein). The off-white background is to differentiate columns from one another. Top Row: scatter plots showing the results of dimensionality reduction performed on primary RNA, mature RNA, and protein expression data using the UMAP algorithm. Each point is a sample, and colors differentiate LIV samples (pink) from PM samples (blue). The horizontal axis is the first UMAP dimension (“UMAP 1”), the vertical axis is the second UMAP dimension (“UMAP 2”). Middle Row: boxplots showing distributions of UMAP 1 values (horizontal axis) stratified on the vertical axis both by LIV-PM status and Parkinson’s disease (PD) status of the samples (LIV samples – pink, PM samples – blue; samples from individuals with PD – A [“Affected”], samples from individuals without PD – U [“Unaffected”]). For each boxplot: the colored line inside the box is the media; the left and right edges of the box are the first and third quartiles (the 25th and 75th percentiles); the right whisker extends from the right edge of the box to the largest y-axis value no further than 1.5 times the interquartile range away from the right edge of the box; the left whisker extends from the left edge of the box to the smallest y-axis value no further than 1.5 times the interquartile range away from the left edge of the box. Bottom Row: scatter plots showing the results of differential expression analysis comparing LIV samples to PM samples for each primary RNA transcript, mature RNA transcript, and protein feature quantified for the bulk RNA-seq and bulk LC-MS analyses in the current report. Each point is a feauture (i.e., RNA transcript or protein). The horizontal axis shows the average normalized RNA transcript or protein expression level. The vertical axis shows the logFC values, and the range of values is −4.95 to 5.29 for primary RNA transcripts, −7.71 to 5.40 for mature RNA transcripts, and −1.71 to 1.27 for protein. Positive logFC values are indicative of higher levels in PM samples compared to LIV samples, and negative logFC values are indicative of higher levels in LIV samples compared to PM samples. Colors differentiate features with levels that were significantly higher in PM compared to LIV (blue), significantly higher in LIV compared to PM (pink), or not significantly different between LIV and PM (gray). (B) RNA and protein LIV-PM DE signature concordance. Scatter plot showing the concordance of RNA transcript and protein LIV-PM DE signatures. Each point is a feature, and only features present in all 3 LIV-PM DE signatures (i.e., primary RNA, mature RNA, protein) are plotted. The x-axis shows the logFC values from the primary RNA transcript LIV-PM DE analysis. The y-axis shows the logFC values from the mature RNA transcript LIV-PM DE analysis. The color of points indicates the logFC values from the protein LIV-PM DE signature. The colors range from pink (greater expression in LIV samples compared to PM samples) to white (no difference in expression between PM samples and LIV samples) to blue (expression greater in PM samples relative to LIV samples). (C) LIV-PM differential splicing rate analysis. An alluvial plot showing changes in the RNA transcript LIV-PM DE signature across three variables (represented by three “pillars” on the horizontal axis) that were tested for differences between LIV and PM samples on the same set of features: levels of primary RNA transcript expression (left pillar), the rate of conversion of primary RNA to mature RNA (middle pillar), and levels of mature RNA expression (right pillar). There are three categories, called “stratum”, within each of the three pillars: RNA features tested that were greater in LIV samples compared to PM samples (pink), greater in PM samples compared to LIV samples (blue), or not significantly different between LIV samples and PM samples (gray). Each “alluvial fan” (i.e., a set of thick wavy lines connecting pillars pillars) shows change of a set of features across the three variables and is comprised of two “flows”, which are the segments connecting adjacent pillars. Flows are colored by the DE status of the stratum from which it originated, and fans can therefore be comprised of flows of multiple colors. The pie charts above each pillar represent the cumulative proportion of the transcriptome that is different between LIV samples and PM samples in at least one of the three levels tested (totaling over 95% as indicated by the proportion of the pie that is colored purple in the last pie chart). (D) LIV-PM differential intron usage example. Differential intron usage between LIV samples and PM samples is shown for the *RSRP1* intron cluster. Exons are represented by the black segments beneath the continuous black line (which represents the full gene). Introns in the cluster are represented by curves that connect two exon ends. The thickness of each curve represents the intron usage ratio. To calculate the intron usage ratio, the mean intron usage was calculated separately for LIV and PM samples and the larger mean intron usage was divided by the smaller mean intron usage. Curves colored pink are introns with greater usage in LIV compared to PM and curves colored blue are introns with greater usage in PM compared to LIV.

DE of LIV-PM status (“LIV-PM DE”) was performed utilizing RNA transcript expression data from 518 PFC samples characterized using bulk RNA-seq (275 LIV samples and 243 PM samples) and protein expression data from 488 PFC samples characterized using bulk LC-MS (248 LIV samples and 240 PM samples; 472 of which were in the 518 samples with bulk RNA-seq data). LIV-PM DE was performed separately for primary RNA transcripts (22,955 features), mature RNA transcripts (30,099 features; 20,671 with primary RNA transcripts also detected), and proteins (6,415 features), and the three resulting LIV-PM DE signatures with corresponding biological pathway enrichment test results are provided in S1 and S2 Table. By convention for this report for LIV-PM DE signatures, positive logFC values represent higher expression in PM samples compared to LIV samples and negative logFC values represent higher expression in LIV samples compared to PM samples. LIV-PM DEFs (i.e., DEFs identified in LIV-PM DE analyses) with negative logFC values are referred to as LIV DEFs and LIV-PM DEFs with positive logFC values are referred to as PM DEFs. Significant differences in expression were observed between LIV samples and PM samples for 74% of primary RNA transcripts (8,892 LIV DEFs and 8,102 PM DEFs), 70% of mature RNA transcripts (13,313 LIV DEFs and 7,817 PM DEFs), and 61% of proteins (1,898 LIV DEFs and 2,003 PM DEFs) (Fig 2A). A significant positive Spearman’s correlation coefficient was observed when comparing the LIV-PM DE signatures from (1) primary and mature RNA transcripts (ρ = 0.45, p-value < 2 x 10^-16^) and (2) mature RNA transcripts and proteins (ρ = 0.18, p-value < 2 x 10^-16^) (Fig 2B).

To establish that the LIV-PM DE signatures identified in this section are not explained by variables with the potential to confound measures of RNA transcript or protein expression, analyses were performed assessing the stability of the LIV-PM DE signatures with respect to the following 12 potential confounding variables: (1) data generation batch; (2) institution of origin of the PM samples; (3) postmortem interval (PMI); (4) diagnosis of PD in living participants and postmortem donors; (5) severity of PD symptoms in living participants; (6) dose of dopamine replacement therapy in living participants; (7) neuropathology in LIV and PM samples; (8) type and dose of anesthesia administered to living participants during DBS surgery; (9) method of LIV sample preservation upon collection during DBS surgery; (10) RNA integrity number (RIN); (11) age differences between living participants and postmortem donors; (12) cell type composition differences between LIV samples and PM samples. The LIV-PM DE signatures were stable with respect to all 12 potential confounding variables, and an extended presentation of these analyses is in the S1 File.

### Splicing rates differ between LIV and PM samples

RNA splicing is the processing of primary RNA into mature RNA. For a given RNA transcript at a single moment in time, the splicing rate (i.e., the amount of mature RNA transcripts relative to the amount of primary RNA transcripts) is an emergent property of transcriptional regulation [11]. Differential splicing rate analysis (i.e., comparing the splicing rate in LIV samples to the splicing rate in PM samples for each RNA transcript expressed) was performed using a regression model that tested the association between LIV-PM status and splicing rates for the 20,671 RNA transcripts with both primary RNA and mature RNA transcript expression detected. Significantly different splicing rates were found for 73% of the RNA transcripts tested (9,448 with greater splicing rates in LIV samples compared to PM samples and 5,641 with higher splicing rates in PM samples compared to LIV samples). These included nearly all of the LIV-PM DEFs that had opposite directions of effect in the primary and mature RNA transcript LIV-PM DE signatures (e.g., RNA transcripts that were LIV DEFs in the primary RNA and PM DEFs in the mature RNA; Fig 2C). For 1,074 RNA transcripts, splicing rates were higher in PM samples compared to LIV samples even though the primary and mature RNA transcript expression levels were higher in LIV samples compared to PM samples (i.e., the RNA transcript was a LIV DEF in both the primary and mature RNA transcript LIV-PM DE signatures). Similarly, for 1,635 RNA transcripts, splicing rates were higher in LIV samples compared to PM samples even though the primary and mature RNA transcript expression levels were higher in PM samples compared to LIV samples (i.e., the RNA transcript was a PM DEF in both the primary and mature RNA transcript LIV-PM DE signatures). Altogether, 95% of the 20,671 RNA transcripts with both primary RNA and mature RNA transcript expression detected significantly differed between LIV samples and PM samples with respect to either primary RNA transcript expression levels, mature RNA transcript expression levels, or splicing rates (Fig 2C).

### Intron usage rates differ between LIV and PM samples

For an intron in a RNA transcript, the intron usage level is the percentage of mature RNA transcripts that result from splicing the intron out of primary RNA transcripts. For a primary RNA transcript containing multiple introns, using different combinations of introns results in different forms of mature RNA transcripts (“isoforms”), which are translated into different forms of the same protein [12]. For a set of introns in a primary RNA transcript (i.e., an intron cluster), differential intron usage analysis tests whether two groups of samples (e.g., LIV samples and PM samples) differ with respect to patterns of intron usage (and, therefore, patterns of mature RNA transcript isoform abundance) [13]. Intron usage levels were quantified for LIV samples and PM samples from the bulk RNA-seq data (for these analyses, a single LIV sample was retained per living participant for the data to be compatible with the software used; Fig 1B). After data processing and quality control procedures had been completed, 11,222 intron clusters (covering 28,001 introns and mapping to 6,797 unique RNA transcripts) were tested for differential intron usage between LIV samples and PM samples. Significant differences in intron usage were detected for 64% of the intron clusters tested (7,141 intron clusters), which covered 66% of the introns tested (18,428 introns; 9,649 introns with higher usage rates in LIV samples and 8,779 introns with higher usage rate in PM samples) and 74% of the RNA transcripts tested (4,972 RNA transcripts; 97% of which significantly differed between LIV samples and PM samples in either the primary RNA transcript, mature RNA transcript, or splicing rate LIV-PM DE signatures). Differential intron usage was most significant for *RSRP1* (adjusted p-value = 5.91 x 10^-162^; Fig 2D), which encodes a component of the spliceosome [14].

### RNA-protein co-expression patterns differ between LIV and PM samples

**Fig 3:**
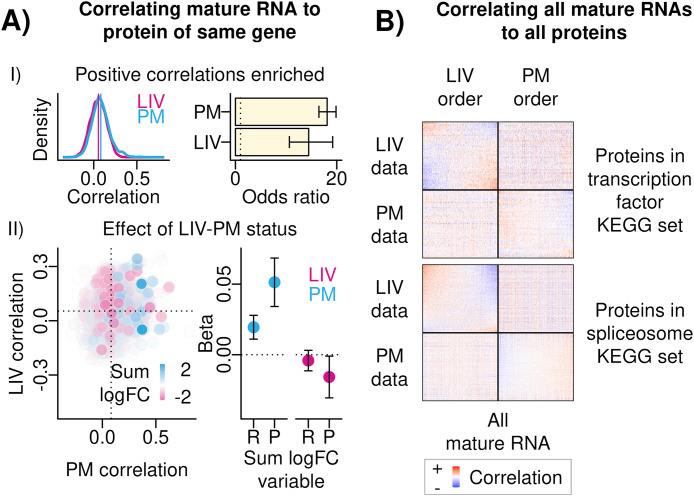
(A) Correlating mature RNA to protein of same gene. **(I)** *Positive Correlations Enriched.* Left – density plots of the same-gene RNA-protein correlations in LIV samples (pink) and PM samples (blue). Right – Bar plot showing the results of tests of whether the same-gene RNA-protein correlations were significant and positive more than expected by chance in LIV samples and PM samples (vertical axis). The summary statistic of this test is the odds ratio, presented on the horizontal axis. Error bars are the 95% confidence interval of the odds ratio from Fisher’s exact test, and error bars that do not cross the dotted vertical line intersecting the horizontal axis at 1 indicate statistical significance. **(II)** *Effect of LIV-PM status*. Left – scatter plot showing relationship of same-gene RNA-protein correlations in PM samples (x-axis) to same-gene RNA-protein correlations in LIV samples (y-axis). To color and shade the points, the mature RNA logFC and the protein logFC were summed; if the sum was greater than 0 the point was colored blue, if the sum was less than 0 the point was colored pink, and the shade of the points was set to reflect the absolute value of the sum. Right – To determine if LIV-PM status was driving same-gene mature RNA-protein correlations, betas (y-axis) and associated p-values were obtained using the following linear model applied separately to LIV samples and PM samples (x-axis): same-gene correlation coefficient ~ Mature RNA LIV-PM logFC (resulting beta is “R” on x-axis) + Protein LIV-PM logFC (resulting beta is “P” on x-axis). **(B)** Correlating all mature RNAs to all proteins. For the two KEGG sets most enriched for differentially correlated RNA-protein pairs (transcription factors; spliceosome) the RNA-protein correlations are shown between the proteins in the set (heatmap rows) and all mature RNA transcripts (heatmap columns). Positive correlations are in red, negative correlations are in blue. “LIV Data” and “PM Data” describe the set of samples (i.e., LIV samples or PM samples) used to generate the correlations in the corresponding row of heatmaps. “LIV Order” and “PM Order” describe the data that was used to order the proteins and mature RNA transcripts in the corresponding column of heatmaps.

RNA-protein co-expression refers to the relative expression levels of RNA transcripts to proteins in a set of tissue samples. A series of analyses were performed on the paired bulk RNA-seq and bulk LC-MS data to test the hypothesis that LIV and PM samples may have distinct patterns of RNA-protein co-expression. To define RNA-protein co-expression, each of the 30,099 mature RNA transcripts was correlated with each of the 6,415 proteins separately in LIV samples and in PM samples. This resulted in two RNA-protein co-expression matrices (i.e., one from LIV samples and one from PM samples) that each contained 193,085,085 RNA-protein correlation values (i.e., one value for every possible pairing of a mature RNA transcript with a protein [“RNA-protein pair”]). Each RNA-protein correlation value was either a “same-gene RNA-protein correlation” (i.e., a correlation between a mature RNA transcript and a protein encoded by the same gene; N = 5,714) or an “different-gene RNA-protein correlation” (i.e., a correlation between a mature RNA transcript encoded by one gene and a protein encoded by a different gene). After multiple test correction, a statistically significant correlation in either the LIV samples or the PM samples was observed for 2,208,798 RNA-protein pairs. The median value of same-gene RNA-protein correlations was 0.06 in LIV samples and 0.07 in PM samples (Fig 3A–I, ***Left Panel***), median values that are consistent with observations from previous studies of postmortem human brain tissue [15] and other human tissue types [16–18]. For both LIV samples and PM samples, same-gene RNA-protein correlations were more likely to be (1) positive and (2) statistically significant compared to different-gene RNA-protein correlations (Fisher’s exact test ORs: in LIV samples = 14.46, in PM samples = 18.11; p-values < 2 x 10^−16^; Fig 3A–I*, Right Panel*), suggesting same-gene RNA-protein co-expression patterns are consistent with expectations from the central dogma of biology (i.e., positive and significant correlations) in both LIV samples and PM samples. The concordance between the same-gene RNA-protein correlations observed in LIV samples and the same-gene RNA-protein correlations observed in PM samples (Pearson’s correlation coefficient = 0.12; Fig 3A ***- II, Left Panel***) suggested that same-gene RNA-protein correlations may be partially explained by LIV-PM status. To test this hypothesis, a linear model was run separately for LIV samples and for PM samples where the same-gene RNA-protein correlations were the dependent variable and the logFC values from mature RNA transcript and protein LIV-PM DE signatures were the independent variables. Both the mature RNA transcript LIV-PM DE logFC values and the protein LIV-PM DE logFC values were significantly associated with the same-gene RNA-protein correlations in PM samples (t-statistics and adjusted p-values: mature RNA LIV-PM DE logFC values = 4.52, 2.47 x 10^−5^; protein LIV-PM DE logFC values = 5.86, 1.93 x 10^−8^) but not in LIV samples (Fig 3A ***- II***).

The differences observed between LIV samples and PM samples with respect to same-gene RNA-protein correlations led to the hypothesis that RNA-protein co-expression more broadly (i.e., same-gene and different-gene RNA-protein correlations) may have a distinct pattern in LIV and PM samples. A LIV-PM correlation difference matrix was calculated by subtracting the LIV sample RNA-protein correlation matrix from the PM sample RNA-protein correlation matrix and transforming the differences to absolute values. For each RNA-protein pair in the LIV-PM correlation difference matrix, an empirical p-value was calculated to assess whether the correlation difference observed between LIV samples and PM samples was greater than expected from 10,000 permutations where two groups that each contained a randomly selected mixture of LIV samples and PM samples were compared. The RNA-protein pairs that had significantly different correlations between LIV samples and PM samples (“differentially co-expressed RNA-protein pairs”) were enriched for the 2,208,798 RNA-protein pairs that were significantly correlated in either LIV samples or PM samples (72.8% of these 2,208,798 RNA-protein pairs were differentially co-expressed; Fisher’s exact test OR = 31.91, p-value < 2 x 10^-16^).

The primary function of certain protein families is to regulate the levels of RNA transcripts (e.g., transcription factors). Analyses were therefore performed to test the hypothesis that differentially co-expressed RNA-protein pairs are enriched for proteins in these families. To test this hypothesis, for 36 protein families defined in the Kyoto Encyclopedia of Genes and Genomes (KEGG) database (which groups genes into sets based on curated annotations from public resources and published literature [19]) a Fisher’s exact test was performed to test whether the protein members of differentially co-expressed RNA-protein pairs are enriched in the KEGG protein family. After multiple testing correction, significant enrichments were seen for 18 of the 36 KEGG protein families evaluated (S3 Table), including protein families responsible for regulating both RNA transcription and RNA splicing (‘transcription factors’ family OR = 1.28, ‘spliceosome’ family OR = 1.43; adjusted p-values < 2 x 10^-16^) (Fig 3B).

## Discussion

Knowledge of how living and postmortem human brain tissues differ at the molecular level is needed to inform the design and interpretation of studies that only utilize postmortem tissue to understand the molecular basis of brain health. Prior to the LBP, studies that have compared living and postmortem brain samples at the molecular level focused on RNA transcript expression. These studies were small in scale, conducted prior to the advent of next-generation sequencing technologies, or limited to comparisons of living and postmortem cohorts not matched for key clinical and technical variables [3–6]. LBP studies performed in parallel to the LBP study reported here also focused on RNA transcript expression, were able to overcome several of the study design limitations of earlier efforts, and identified widespread differences in RNA transcript expression, co-expression, and editing [9,20,21]. By applying different analytic strategies to the same bulk RNA-seq data analyzed in the other LBP studies, as well as by integrating that data with proteomic data introduced here, the study reported here finds that differences are also detected at the levels of RNA splicing and protein expression. Specifically, significant differences between living and postmortem PFC samples were observed with respect to (1) the expression of most primary RNA transcripts, mature RNA transcripts, and proteins, (2) the splicing of most primary RNA transcripts into mature RNA transcripts, and (3) the patterns of co-expression between RNA transcripts and proteins. In addition to finding many molecular differences between living and postmortem samples, this study also found some key similarities. For example, the same-gene RNA-protein correlations were low both in LIV samples and in PM samples.

The study had several limitations. The current study was not designed to dissect the molecular mechanisms that give rise to the LIV-PM DE signatures identified. To do so will require studies of model systems (e.g., cell lines, rodents), and those studies should seek to establish the extent to which the procedures used to acquire and process PFC biopsies from living participants could impact RNA transcript and protein expression. Another limitation of the study design is that while RNA-seq and LC-MS are state-of-the-art technologies for characterizing the transcriptome and proteome in human tissues, respectively, these technologies remain limited in their ability to fully capture the molecular foundations of tissue function [16–18]. This is evident in some of the study results, such as the low same-gene RNA-protein correlations observed both in LIV samples and in PM samples. When these technologies are succeeded by improved technologies in the future, studies should aim to refine the observations made here. Additional limitations include: not all potential confounding variables could be accounted for in DE analyses, nor could all possible interactions between potential confounding variables be considered.

Altogether, the findings of the current report show that depending on the research question of interest, the molecular differences that exist between living and postmortem human brain tissues may or may not matter, and future work should aim to determine which research objectives can and cannot be adequately achieved using only postmortem human brain tissue.

## Methods

### Ethics statement

The study was approved by the Human Research Protection Program at the Icahn School of Medicine at Mount Sinai. All human subjects research was carried out under STUDY-13–00415. Research participants in the living cohort provided verbal and written informed consent for sample collection, genomic profiling, clinical data extraction from medical records, and public sharing of de-identified data. All the methods were performed in accordance with the relevant guidelines and regulations. Recruitment for STUDY-13–00415 began on August 5^th^, 2013 and is ongoing at the time of writing.

### Living brain project cohort

All of the individuals and PFC samples studied in the current report were first introduced in an earlier LBP report [8]. In the methods sections of that report can be found detailed descriptions of the living cohort, the postmortem cohort, PFC sample collection procedures, and clinical data collection (i.e., anesthesia given to the living cohort, PD symptom severity in the living cohort, and dopamine replacement therapy in the living cohort).

### Bulk RNA-seq

In this section, the methods used to perform bulk RNA-seq and prepare the resulting bulk RNA-seq data for analysis are presented. Towards this end, four “pipelines” (where a pipeline is defined as a sequential series of steps that together achieve a specific goal) were implemented:

1) RNA Sequencing Pipeline: the goal of this pipeline is to generate the bulk RNA-seq data for analysis.
2) Confounder Identification Pipeline: the goal of this pipeline was to identify unwanted drivers of variance in RNA transcript expression to include as covariates in bulk RNA-seq data analyses.
3) Primary and Mature RNA Quantification Pipeline: the goal of this pipeline was to concurrently quantify the levels of primary and mature forms of each RNA transcript detected in the bulk RNA-seq data.
4) Intron Usage Quantification Pipeline: the goal of this pipeline was to quantify intron usage in bulk RNA-seq data.

#### RNA Sequencing Pipeline.

The methods used to perform RNA extraction, RNA sequencing, cell type deconvolution, and data quality control for the bulk RNA-seq data analyzed in the current report are introduced and fully described in Liharska *et al.*[8] and briefly summarized here. Approximately 5–10 milligrams of each sample was used for RNA extraction. Extraction was generally performed in batches of 12 samples using the RNeasy Kit (Qiagen, Hilden, Germany), mostly according to manufacturer instructions. Only specimens with an RNA integrity number (RIN) greater than 4.0 were sent for RNA sequencing. Preparation of cDNA libraries and RNA sequencing were performed at Sema4 (Stamford, CT). Libraries of cDNA were prepared using the TruSeq Stranded Total RNA with Ribo-Zero Globin Kit (Illumina, San Diego, CA; Catalog Number 20020613). RNA sequencing was performed on the NovaSeq 6000 System (Illumina, San Diego, CA). Base calls were made from data emitted by the clusters within the S4 flow cell and organized into sequencing reads stored in FASTQ files using Illumina’s bcl2fastq software (v1.8.4).

#### Confounder Identification Pipeline Summary.

The methods used to identify confounders to account for in the bulk RNA-seq data analyzed in the current report are introduced and fully described in Liharska *et al*. [8] and briefly summarized here. Unaligned reads were aligned to the GRCh38 primary assembly with Gencode gene annotation v30 using STAR (v2.7.2a) [22,23]. Aligned reads were sorted using samtools (v1.13) [24] and duplicate reads were marked using Picard Tools (v2.20.1). Unwanted drivers of variance in RNA transcript expression to include as covariates in bulk RNA-seq data analyses were identified using an iterative pipeline that considered over one hundred technical variables.

#### Primary and Mature RNA Quantification Pipeline.

FASTA and General Transfer Format (GTF) source files for the human genome GRCh38 primary assembly with Gencode gene annotations v41 were obtained from the Gencode website. (https://ftp.ebi.ac.uk/pub/databases/gencode/Gencode_human/release_41/) [23]. The GTF file was input into the getFeatureRanges() function of the eisaR R package (v1.8.0) with the featureType parameter set to “spliced” (which corresponds to mature RNA) and “unspliced” (which corresponds to primary RNA) and default settings used for the other parameters [25]. With these settings, the getFeatureRanges() function parses the input GTF to return an object that for each transcript in the GTF annotated in the GTF as having >=1 exon there are two entries: one that provides a genomic range for each exon (i.e., its start and stop positions) and one that contains a single genomic range for the transcript that is inclusive of all the exon ranges as well as the genomic ranges separating the exon ranges (i.e., the intronic ranges). The getTx2Gene() of the eisaR R package was then applied to this object to create a file linking transcript identifiers to gene identifiers that was then used in subsequent steps described below. The GRCh38 FASTA file was then read into R using the readDNAStringSet() function of the Biostrings R package (v2.60.1). The resulting object, along with the object output by the getFeatureRanges() function, were then input into the extractTranscriptSeqs() function of the GenomicFeatures R package (v1.44.0). This function iterates over each entry in the object output by the getFeatureRanges() function and extracts the genomic sequence corresponding to the ranges in the entry. For entries that correspond to mature RNA, the resulting sequence is a single string of the combined exonic sequences. For entries that correspond to primary RNA, the resulting string contains the full transcript sequence (i.e., exons and introns). The resulting set of sequences were then saved as a FASTA file using the writeXStringSet() function of the Biostrings R package. The newly created FASTA was then input into the index function of the salmon unix package (v1.9.0) along with the full reference FASTA from which it was created. The two FASTA files were supplied as the “--transcripts” argument to the index function along with the “--gencode” flag (specifying the FASTA files are in Gencode format) and a list of all sequence names in the full reference FASTA (e.g., chromosome names) to the “--decoys” argument [26] and otherwise default parameters [26]. The output of this step was a salmon index (“SIDX”) file.

For each of the 518 samples with bulk RNA-seq data the salmon quant command was run giving as input the FASTQ files containing the RNA sequencing reads for the sample (the “--mates1” and “--mates2” arguments), the SIDX file (to the “--index” parameter), “--libType” set to ISR, and otherwise default settings. From the resulting set of quantification files gene-level counts were derived for each sample using the tximport() function of the tximport R package (v1.20.0) with the “type” parameter set to “salmon” and the “countsFromAbundance” parameter set to “lengthScaledTPM” and the “tx2gene” parameter set to a path to a file linking transcript to gene identifiers created as described above during the preparation of the SIDX file [27,28]. Separate counts matrices were created for primary RNA (38,387 features) and mature RNA features (61,436 features; 38,344 overlapping with primary RNA). Lowly expressed RNA features were filtered out by calculating the log of the mean of the gene-level TPM (calculated by summing the transcript-level TPM values output by salmon quant). Any gene with a value less than negative five was removed, leaving 22,955 primary RNA and 30,099 mature RNA features for analysis. The counts of these features output by tximport were normalized using the voomWithDreamWeights function of the dream software within the variancePartition R package (v1.20.0) because two samples per individual were available for some individuals [29,30].

#### Intron Usage Quantification Pipeline.

The “junction extract” command of the regtools unix package (v0.5.1) was run to quantify the usage of each intron based on CIGAR strings from the BAM files generated as described above [31]. Default settings were used with the exception that the minimum intron length (“-m”) was set to 50 and the strand specificity (“-s”) was set to 2.

The file output by regtools, which contains one row per intron and the corresponding intron usage for the sample, was then used as the input to LeafCutter (v0.2.9) in order to identify intron clusters [13]. The purpose of the intron clustering step in LeafCutter is to represent as a unit different forms of intronic excisions that occur on the same region. LeafCutter defines intron clusters in a manner such that every intron in the cluster must share a start or stop position with at least one other intron in the cluster. When represented as a graph where each intron is an edge and intron start and stop positions are vertices, an intron cluster is a connected graph (i.e., there is a path between all vertex pairs). Intron clusters were identified using the LeafCutter “leafcutter_cluster_regtools.py” script, which was given as input the regtools output for all samples together (N = 518) and settings such that 50 split reads were required to support the definition of an intron cluster and introns spanning up to 500,000 base pairs in size were considered. The output of this script was a matrix of counts with one row per intron (i.e., multiple rows per intron cluster) and one column per sample.

A series of filtering steps were then applied to either introns or intron clusters in the output of the LeafCutter intron clustering step, adapted from a previous study on human aging [32]. For each intron, the percentage of samples with 0 counts was calculated and any intron with>=25% of the samples having counts of 0 were flagged for removal. For each intron cluster, the contribution of each intron to the cluster was calculated as follows: the counts for the intron were calculated by summing the counts for the intron across all samples; the counts for the intron cluster were calculated by summing the counts for all the introns in the cluster; the contribution of the intron to the cluster was the calculated as the intron counts divided by the intron cluster counts. Any intron with a contribution less than 5% was flagged for removal. After applying these intron filters, each intron cluster in the output was then reassessed to determine if the criteria used by LeafCutter to define intron clusters was still fulfilled – in other words, to determine that the cluster still was a connected graph when represented as a graph with introns as edges and intron start and stop positions as vertices. For a given intron cluster, there are three possible outcomes to this reassessment: (1) the intron cluster is found to have a single edge, in which case it is removed; (2) the intron cluster remains a connected graph, in which case it is retained; (3) the intron cluster is found to have multiple introns but is no longer a connected graph. In the event of the latter, the intron cluster was decomposed into subgraphs of connected components. If all subgraphs have a single intron, the intron cluster is removed. If any subgraphs have greater than one intron, a “pruning” procedure was performed on the intron cluster. The pruning procedure consists of decomposing the intron cluster to its connected subgraphs and retaining the subgraphs with the most introns (i.e., if there are three subgraphs and they have 2, 2, and 1 intron, the subgraphs with 2 introns will be both be retained and the subgraph with 1 intron will be pruned). If more than one subgraph is retained by this procedure, those subgraphs are split and considered separate intron clusters moving forward. After the pruning step, the number of introns in each intron cluster remaining was calculated and only intron clusters with 2–10 introns were retained.

### Bulk liquid chromatography-mass spectrometry (LC-MS)

#### Protein extraction and digestion.

PFC samples were lysed with a lysis buffer containing 8M Urea, 50mM Tris-HCl pH 8.0, 1% Protease and Phosphatase Inhibitor Cocktail, and Optima LC-MS Water. Lysis buffer (200 μL) was added to each sample and mixed by pipetting up and down, and then the whole sample was immediately transferred out of the sample vial and into a 1.5mL Eppendorf tube. Each sample was sonicated with four three-second pulses at 20% amplification to fully lyse the cells. Sonicated samples were centrifuged at 17,000 x g for 10 minutes, and the supernatant was then used in the Bradford Assay to determine the protein concentration. Proteins were reduced with 10mM Tris(2-carboxyethyl) Phosphine (TCEP) and alkylated with 18.75mM iodoacetamide Samples were diluted 1:5 with deionized water and digested with sequencing grade modified trypsin (Promega, Madison, USA; Catalog Number V5111) at a 1:50 enzyme-to-substrate ratio.

#### Peptide labeling.

TMT (Tandem Mass Tag) reagents (16-plex; one “plex” labels all peptides from one sample so that peptides from multiple samples can be pooled together into a “multiplex”) were used to label peptides from all samples in 34 batches of pooled peptides (from 14–15 samples per batch), allowing relative quantitation (i.e., estimation of relative quantity) (Thermo Fisher Scientific, Waltham, USA; Catalog Number A44520). For tissue samples, a reference sample was created by pooling an aliquot of peptides from each individual sample. Peptides (20 µg) from each of the samples were dissolved in 20 µL of 200 mM triethylammonium bicarbonate (TEAB), pH 8.5 solution, and mixed with 20 µL of TMT reagent that was freshly dissolved in 256 µL of anhydrous acetonitrile, LC-MS grade. Channel 126 was used for labeling the pooled reference sample in all matrices analyzed in this study. After 4 hours of incubation at room tempterature, the reaction was quenched by adding 8 µL 5% hydroxylamine. Peptides labeled by different TMT reagents were then mixed, dried and desalted on C18 Spin columns. Desalted peptides were dried in a vacuum centrifuge and stored at −20°C until LC-MS. For technical reasons, it was not possible for each batch of pooled peptides to be comprised of equal numbers of LIV samples and PM samples.

#### Peptide fractionation.

The newly formed batches were fractionated using basic reversed-phase liquid chromatography. Approximately 160 μg of 16-plex TMT labeled sample was first purified on C18 column, and then separated on a reversed phase Zorbax extend-C-18 column (4.6 × 100 mm column containing 1.8-um particles; Agilent, Santa Clara USA) using an Agilent 1200 Infinity HPLC System (Agilent, Santa Clara, USA). The solvent A consisted of 10 mM ammonium formate, pH 10.0. Solvent B consisted of 10 mM ammonium formate, pH 10, 90% acetonitrile as mobile phase. The separation gradient was set as follows: 2% B for 10 min, from 2 to 16% B for 10 min, from 16 to 40% B for 65 min, from 45 to 95% B for 5 min, and 95% B for 15 min. A total of 96 fractions were collected into a 96 well plate in a time-based mode. These fractions were then concatenated into 24 fractions by combining 4 fractions that are 24 fractions apart (i.e., combining fractions #1, #25, #49, and #73; #2, #26, #50, and #74; and so on). Each concatenated fraction was dried down in a Speed-Vac and re-suspended in 2% acetonitrile, 0.1% formic acid for LC-MS.

#### Mass spectrometry.

LC-MS was performed using a Waters nanoAcquity LC (Waters, Milford, USA) system coupled to a Thermo Q Exactive Plus MS (Thermo Fisher Scientific, Waltham, USA). Peptides were separated in a 90 minute gradient from 5% B to 35% B. The eluting peptides were sprayed into the mass spectrometer using electrospray ionization and a data dependent Top 15 acquisition method was used to fragment candidate ions. Full MS survey scans were collected at a resolution of 35,000, scan range of 400–1800 Thompsons (Th; Th = Da/z), followed by MS/MS scans at a resolution of 35,000 with a 1.2 Th isolation window. Only ions with a + 2 to +5 charge were considered for isolation and fragmentation. Data was searched using Proteome Discoverer 2.5 (Thermo Fisher Scientific, Waltham, USA) using SEQUEST algorithms and RefSeq database.

#### Data normalization, imputation, and quality control.

Log2 transformed sample-to-reference ratio of MS2 (log2-ratio) intensity was produced for proteomics from 510 samples. In total 10,515 proteins were identified from the experiment, among which 2,310 were completely observed among all samples, and the overall missing rate was 39.7%. To preprocess the log2-ratio intensity data matrix, the procedure described in Wang *et al.*, 2021 was followed [33]. To remove the technical variation among the sample distribution globally, sample median alignment to log2-ratio intensity matrix was performed. For each protein, an “inter-TMT-multiplex t-test” was performed (between the log2-ratio of samples inside the TMT multiplex and the log2-ratio of samples outside of the TMT multiplex) to detect and remove outlier TMT multiplex values for the protein. For each protein, after double log-transformation, p-values of “inter-TMT-multiplex t-test” falling beyond four standard deviations from the median of the entire dataset were flagged as outliers, and the corresponding values of the specific protein for the respective TMT multiplex were labeled as “N/A” in the dataset. In total, outlier TMT multiplex values for six individual proteins were removed from the dataset (corresponding to six distinct TMT multiplexes for which all data for one respective protein was removed). Following outlier removal, the ComBat algorithm [33] was applied to the dataset to correct for the effect of the TMT multiplex batch variable. In order to preserve the effect of LIV-PM status (which was correlated with TMT multiplex batch), the LIV-PM status variable was included in the ComBat model in addition to the TMT multiplex batch variable. Based on the resulting model values, only the calculated effect of TMT multiplex batch was regressed out of the protein expression data, while the calculated effect of LIV-PM status was preserved (i.e., protein expression was calculated as the sum of the effect of LIV-PM status and the residuals). Due to the ComBat algorithm’s requirement for complete data, KNN imputation was performed on the data prior to application of the ComBat algorithm using the impute R package [34]. After the imputed data was corrected using ComBat [35], the missing data structure from before KNN imputation was restored. To formally impute the missing values, the DreamAI software was applied [36]. Imputation was done for the subset of 6,415 proteins that appeared in at least 50% of samples.

In order to identify unwanted drivers of variance to include as covariates in statistical models of protein expression, technical metrics and batch assignments characterizing the protein expression data were merged with the table of covariates characterizing the individuals (e.g., age, sex) and samples of the dataset. Covariates explaining the variance in protein expression between samples were then reviewed for potential inclusion in the statistical model of the data used for downstream analyses through an adaptation of an iterative procedure established in Liharska *et al.* [21] for bulk RNA-seq data. First, LIV-PM status and individual ID were fit to a linear mixed model with individual ID as a random effect using the dream() function of the variancePartition R package, and residuals were calculated with the residuals() function of the base stats R package. Individual ID was accounted for as data from more than one sample per individual was available for some living participants. Accounting for the effect of LIV-PM status on the variance in protein expression at this step allowed for the potential effects of other covariates on the protein expression variance to subsequently be observed. Next, principal component analysis (PCA) was performed on the residual protein level matrix using the prcomp() function of the base stats R package and the canonical correlation between the variables in the covariate table and principal components (PCs) 1–5 of the residual RNA level data was calculated using the canCorPairs() function of variancePartition. Scatterplots of PCs (e.g., PC1 vs PC2) were generated and colored for each covariate as a visual aid in assessing the strength of the relationships between covariates and RNA levels. Canonical correlations and visual aids were reviewed and one covariate was considered for inclusion in downstream models. Due to the high correlation between LIV-PM status and TMT multiplex batch (canonical correlation = 0.59), and the fact that the data had already been corrected for TMT multiplex batch using the ComBat algorithm to remove the effect of TMT multiplex batch (and accordingly some partial effect of LIV-PM status also accounted for by TMT multiplex batch, as well as effects of any other correlated batch variables), the iterative procedure did not identify any additional covariates for inclusion in the statistical model of the data for downstream analyses. Samples were considered for removal for being outliers in the protein expression data. Outliers were defined as samples falling more than three standard deviations away from the centroid of PC1 and PC2 of the residual protein expression matrix after accounting for covariates selected using the above procedures. No samples were identified for removal.

### Immunohistochemistry

Six types of immunohistochemistry stains were performed on LIV samples and PM samples at the Icahn School of Medicine at Mount Sinai. For all six types of stains, tissue sections with a thickness of approximately 5–10 micrometers (μm) were formalin-fixed, paraffin-embedded, and baked on charged slides at 70–80°C for an average of 15–30 minutes. Scoring of stains was performed by laboratory technician trained by a neuropathologist over the course of months, during which they jointly reviewed a large number of stained slides to establish reliable scoring practices. This process continued until the neuropathologist determined that the technician was demonstrating consistent evaluations across key histopathological features, at which time the technician began scoring independently. In cases of uncertainty, the technician continued to consult the neuropathologist to ensure scoring accuracy.

#### Alpha-synuclein, amyloid, and tau staining.

Chromogenic immunohistochemistry was performed on the BOND Rx Automated Research Stainer (Leica Biosystems, Wetzlar, Germany), as follows:

(1) Heat-induced epitope retrieval was performed at a pH of 9 on the slides for 20 minutes
(2) Slides were then incubated in the primary antibody for 30 minutes. The primary antibody used for Lewy body staining was mouse anti-alpha-synuclein (1:6000 antibody to diluent, LB509, MABN824MI, MilliporeSigma). The primary antibody used for amyloid staining was amyloid β (4G8) (1:4000 antibody to diluent, 4G8, BioLegend, Catalog # 800701). The primary antibody used for tau staining was Phospho-Tau (Ser202, Thr205) Monoclonal Antibody (AT8) (1:2000 antibody to diluent, AT8, Invitrogen, Catalog # MN1020).
(3) The slides were then incubated for 20 minutes with the BOND Polymer Refine Detection kit (DS9800, Leica Biosystems, Wetzlar, Germany), which uses 3,3’-Diaminobenzidine (DAB) chromogen and hematoxylin counterstain to visualize the primary antibody in the tissue section.

Whole slide images were scanned at 40x magnification using the Versa 8 Scanner (Leica Biosystems, Wetzlar, Germany).

#### Luxol hematoxylin and eosin staining.

Before undergoing sequential staining with Luxol Fast Blue and Hematoxylin and Eosin, slides were deparaffinized with the Leica ST5010 Autostainer XL by immersion XYLENE (3 stations, 4 minutes each), 100% EtOH alcohol (2 stations, 3 minutes each), 95% EtOH grade alcohol (2 stations, 3 minutes each), and wash (1 station, 3 minutes). Luxol Fast Blue staining was then done by hand, immersing slides in Luxol Fast Blue solution (1%) at 60°C for 1–2 hours with a tightly capped container then rinsing sequentially with 95% ethanol (1–3 dips), distilled water, Lithium Carbonate Solution (0.1%) (2–3 dips), 95% ethanol (2–3 dips), and tap water. Hematoxylin was applied to the tissue sections for 6 minutes to enhance cellular nuclei, followed by a 2 minute rinse to remove excess stain. A brief 1% Hydrochloric acid solution was applied for 5 seconds in order to enhance staining specificity, followed by a 1 minute wash to remove residual chemicals. Next, a 0.05% ammonia wash was performed for 1 minute, followed by a 1 minute rinse to remove any remaining chemicals. Dehydration of the tissue was accomplished using 95% ethanol for 30 seconds. Eosin was then applied for 3 minutes to stain cytoplasmic structures, followed by an additional tissue dehydration step with 100% reagent alcohol for 3 stations (20 seconds, 30 seconds, 30 seconds). Lastly, the tissue sections underwent clearing with XYLENE for 4 stations, 1 minute each.

#### Bielschowsky silver staining.

Slides were deparaffinized and hydrated in distilled water. The Bielschowsky Silver staining was conducted using the Leica ST5010 Autostainer XL. Two solutions of silver nitrate were prepared, a 20% solution (10 g silver nitrate in 50 mL distilled water) and a 10% solution (10 g silver nitrate in 100 mL distilled water). The 20% silver nitrate solution was heated to 60°C for 15 minutes, then the slides were incubated in the heated solution for 15 minutes. Slides were rinsed in distilled water. A formalin solution was prepared of formaldehyde (37–40%) (2 mL) in distilled water (98 mL). A sodium carbonate solution was prepared by mixing 8g sodium carbonate in 30 mL distilled water. An ammoniacal silver solution was prepared by treating the 10% silver solution with ammonium hydroxide until the precipitate had almost disappeared, followed by adding sodium carbonate solution (0.5 mL) and ammonium hydroxide (25 drops). The formalin solution was added to the ammoniacal silver solution. This solution was poured over slides and developed for 5–30 minutes until golden brown. Slides were rinsed in water and placed in sodium thiosulfate (5 g thiosulfate in 100 mL water) solution for 2 minutes, then washed, dehydrated, cleared, and mounted with synthetic resin.

#### Lewy body scoring (AS).

A laboratory technician blinded to the clinical histories of living participants and postmortem donors reviewed each whole slide image for the presence and density of Lewy bodies. The technician assigned a numerical score to each whole slide image based on the following grading system: 0 (no evidence of Lewy neurites or bodies), 1- (some Lewy neurites, no Lewy bodies), 1 (1 Lewy body), 1+ (2 Lewy bodies), 2- (3–6 Lewy bodies), 2 (7–9 Lewy bodies), 2+ (10–12 Lewy bodies), 3- (13–15 Lewy bodies), 3 (16–18 Lewy bodies), or 3+ (>= 19 Lewy bodies). Scoring criteria were relative to tissue sections of size 1500 μm x 1500 μm and were adjusted for tissue sections of larger or smaller size.

#### Intracellular β-amyloid, cerebral amyloid angiopathy (CAA), and Aβ plaques scoring (4G8).

A laboratory technician blinded to the clinical histories of living participants and postmortem donors analyzed each whole slide image to evaluate the presence and density of intracellular β-amyloid, cerebral amyloid angiopathy (CAA), focal Aβ plaques, and diffuse Aβ plaques. Whole slide images were assigned a final numerical score based on the following grading system: 0 (no evidence of intracellular β-amyloid/angiopathy/Aβ plaques, or white matter only), 1- (intracellular β-amyloid only), 1 (1 instance of angiopathy or plaque, with or without intracellular β-amyloid), 1+ (2–8 instances of angiopathy or plaque, with or without intracellular β-amyloid), 2- (9–15 plaques, with or without intracellular β-amyloid; if there is angiopathy, increase the score value by one), 2 (15–18 plaques, with or without intracellular β-amyloid; if there is angiopathy, increase the score value by one), 2+ (19–25 plaques, with or without intracellular β-amyloid; if there is angiopathy, increase the score value by one), 3- (26–35 plaques, with or without intracellular β-amyloid; if there is angiopathy, increase the score value by one), 3 (>= 36 plaques, with or without intracellular β-amyloid; if there is angiopathy, increase the score value by one), and 3+ (>= 36 plaques and angiopathy, with or without intracellular β-amyloid). Scoring criteria were relative to tissue sections of size 1500 μm x 1500 μm and were adjusted for tissue sections of larger or smaller size.

#### Neurofibrillary tangles (NFTs), glial fibrillary tangles (GFTs), and neuropil thread scoring (AT8).

A laboratory technician blinded to the clinical histories of living participants and postmortem donors reviewed each whole slide image for the presence and density of neurofibrillary tangles (NFTs), glial fibrillary tangles (GFTs), and neuropil threads. Whole slide images were given a final numerical score of 0 (no evidence of NFTs/GFTs/neuropil threads, or white matter only), 1- (neuropil threads only), 1 (1 NFT), 1+ (2 NFTs), 2- (3–6 NFTs, or 1–3 instances of both NFTs and GFTs), 2 (7–9 NFTs, or 4–6 instances of both NFTs and GTFs), 2+ (10–12 NFTs, or 7–9 instances of both NFTs and GFTs), 3- (13–15 NFTs, or 10–12 instances of both NFTs and GFTs), 3 (16–18 NFTs, or 13–15 instances of both NFTs and GFTs), or 3+ (>=19 NFTs, or 16 + instances of both NFTs and GFTs). Scoring criteria were relative to tissue sections of size 1500 μm x 1500 μm and were adjusted for tissue sections of larger or smaller size. Pretangles were not taken into consideration during the scoring process.

#### Gray matter, white matter, and myelin pallor scoring (LHE).

A laboratory technician blinded to the clinical histories of living participants and postmortem donors reviewed each whole slide image for the presence of gray matter, white matter, and myelin pallor. The features were indicated as present or absent using Y/N/NA/X markers. Scoring criteria were relative to tissue sections of size 1500 μm x 1500 μm and were adjusted for tissue sections of larger or smaller size.

#### Neurofibrillary Tangles (NFTs) and Neuritic plaques scoring (Bielschowsky silver).

A laboratory technician blinded to the clinical histories of living participants and postmortem donors reviewed each whole slide image for the presence of Neurofibrillary Tangles (NFTs) and Neuritic plaques. The features were indicated as present or absent using Y/N/NA/X markers. Scoring criteria were relative to tissue sections of size 1500 μm x 1500 μm and were adjusted for tissue sections of larger or smaller size. Non-neuritic amyloid plaques were considered during the scoring process.

### Identity concordance

To identify sample mislabeling events, identity concordance between samples was performed using genetic variants called from the sequencing data analyzed in this report and sequencing data from the same individuals generated for other ongoing LBP studies. More than one data source was available for identity concordance for all but one individual. Variants were called from RNA sequencing data following Genome Analysis Toolkit (GATK) best practices. The approach used to determine if two variant call sets were from the same individual differed depending on the sources of the call sets in the comparison (e.g., RNA sequencing data to RNA sequencing data, whole-genome sequencing [WGS] to RNA sequencing data). For comparisons of (1) two call sets from bulk RNA sequencing data, (2) two callsets from single-cell RNA sequencing data, and (3) two call sets from WGS data, gtcheck from the bcftools software package (v1.9) was used to calculate the percentage of sites concordant between the call sets. For comparisons of (1) bulk RNA sequencing data call sets to single-cell RNA sequencing data call sets, (2) WGS data callsets to bulk RNA sequencing data call sets, and (3) WGS data callsets to single-cell RNA sequencing data call sets, genotyping matrices were read into R, discrepancies between allele code fields were corrected, sites covered in only one call set were removed, and Pearson’s correlations of genotype similarity were calculated for every pair of call sets. Regardless of the approach used to calculate concordance between call sets, a threshold for deciding whether two samples came from the same individual was determined manually by assessing the distribution of similarity metrics. Mismatches were defined as (1) instances where two samples expected to be from the same individual were genetically discordant and (2) instances where two samples not expected to be from the same individual were genetically concordant. All mismatches identified by the thresholding procedure were further examined to confirm a true mismatch (i.e., using all RNA sequencing data and WGS data call sets from the individuals in the mismatch).

### KEGG enrichment tests

Gene set enrichment analyses were performed using the Kyoto Encyclopedia of Genes and Genomes (KEGG) database [19]. The htext-formatted hsa00001 KEGG database was downloaded on October 4th, 2021, via the KEGG web server and parsed into structured data tables in python. The output of this script was a row for every instance of a feature’s membership in a KEGG gene set and one column each for gene symbol, gene set (“KO reference pathway”), parent category of the gene set (“super pathway”), and the broad concept grouping of the parent category (“top-level string”). Gene symbols provided by KEGG were mapped to Ensembl identifiers with a mapping file generated using the HUGO [37] web server on June 10th, 2020. All KEGG gene sets with the top-level string “Human” were excluded from analysis since these gene sets may be derived from studies of postmortem human tissues. Sets with less than 10 member genes were excluded from analysis, leaving 278 KEGG gene sets tested for enrichment. These 278 KEGG gene sets were tested for enrichment of DEFs in R using the fisher.test() function in the base stats package as the overlap between the genes in the KEGG gene set and DEFs, using as background the set of features (i.e., RNA transcripts or proteins) in the DE analysis. KEGG gene sets with statistically significant associations were mapped to parent terms via the source data file. Multiple testing correction was carried out separately for each of the DE signatures investigated accounting for the 278 KEGG gene sets tested using the false discovery rate estimation method of Benjamini and Hochberg [38] implemented in R using the p.adjust() function of the base stats package.

### Comparing DE signatures

Spearman’s correlations between pairs of DE signatures presented throughout this report were calculated using the cor.test() function in the R base stats package. Multiple test correction for these Spearman’s correlations was not performed because the majority of unadjusted p-values returned by the cor.test() function were estimated to be 0 (indicated in the main text using the “p-value < 2.2 x 10^-16^” notation). When throughout this report the overlap between two DEF sets was compared using Fisher’s exact test, the Fisher’s exact test was implemented by the fisher.test() function in the base stats R package.

### Calculating residual bulk RNA-seq and bulk LC-MS expression levels

Residual bulk primary RNA transcript, mature RNA transcript, and protein expression data was calculated using the following formulas applied to the normalized counts using the residuals() function of the base stats R package:

Formula 1 (linear model with fixed effects and random effects for individual ID, sex, and depletion batch):

*RNA Expression ~ Individual ID + Sex + RIN + Neuronal Fraction + Picard RNASeqMetrics Median 3’ Bias + Picard RNASeqMetrics PCT mRNA Bases + Depletion Batch + Picard InsertSizeMetrics Median Insert Size + Picard AlignmentSummaryMetrics Strand Balance (First of Pair) + Residuals*

Formula 2 (linear model with random effects for individual ID):

*Protein Expression ~ Individual ID + Residuals*

After applying these formulas, dimensionality reduction was performed on residual primary RNA, mature RNA, and protein expression data using the Uniform Manifold Approximation and Projection (UMAP) method implemented through the umap() function in the umap R package (v0.2.8.0) with the “n_components” parameter set to 2.

For RNA-protein co-expression analyses, the formula was used to calculate residuals for RNA did not include neuronal cell fraction. As described in the methods section, for both RNA and protein expression data, the process for selecting covariates to include in models consisted of evaluating the effects of each potential covariate on the top PCs of expression data. This process resulted in the neuronal cell fraction estimate being selected for inclusion of RNA data. The neuronal cell fraction is estimated using cell-type-specific references of postmortem human brain gene expression. Such references do not exist for human brain proteomics data. Neuronal cell fraction estimates derived from RNA transcript expression could not be used to estimate neuronal cell fractions from protein expression of the same sample for two reasons: (1) different portions of the sample were used for RNA sequencing and protein, such that the cell type fraction of the portion of the sample used for RNA sequencing cannot be assumed to be representative of the cell type fraction of the portion of the samples used for LC-MS, and (2) the low same-gene correlations between RNA transcript and protein expression from previous studies [15]. Since neuronal cell fraction could only be estimated for one of the data modalities, when performing the analyses integrating the two analyses a decision had to be made regarding whether to include neuronal cell fraction as a covariate in the RNA sequencing data or not. The goal was to make this decision based on what would result in a true representation of the RNA transcript and protein co-expression in the samples, and it was therefore decided to allow cell type-specific effects on expression to remain in the RNA transcript data since these effects could not be accounted for in the protein expression data. Residuals of RNA transcript data for the RNA-protein co-expression analyses were therefore calculated using the following formula:

Formula 3 (linear model with fixed effects and random effects for individual ID, sex, and depletion batch):

*RNA Expression ~ Individual ID + Sex + RIN + Picard RNASeqMetrics Median 3’ Bias + Picard RNASeqMetrics PCT mRNA Bases + Depletion Batch + Picard InsertSizeMetrics Median Insert Size + Picard AlignmentSummaryMetrics Strand Balance (First of Pair) + Residuals*

### LIV-PM DE on bulk RNA-seq and bulk LC-MS data

For each of the LIV-PM DE analyses performed on the bulk RNA-seq and bulk LC-MS data, DE was run on a normalized count matrix using the dream() function in the variancePartition R package and one of the formulas indicated below. For each DE analysis, multiple test correction was done using an FDR of 5%. The LIV-PM DE analyses performed on the bulk RNA-seq and bulk LC-MS data in this report were as follows:

1) Primary RNA LIV-PM DE [Formula 8]
2) Mature RNA LIV-PM DE [Formula 8]
3) Protein LIV-PM DE [Formula 9]

Formula 4 (linear model with fixed effects and random effects for individual ID, depletion batch, and sex):

*RNA Transcript Expression ~ DE Trait + Individual ID + Sex + Percentage Neuronal Fraction + RIN + Picard RNASeqMetrics Median 3 Prime Bias + Picard RNASeqMetrics PCT mRNA Bases + Depletion Batch + Picard InsertSizeMetrics Median Insert Size + Alignment Summary Metrics + Residuals*

Formula 5 (linear model with fixed effects and random effects for individual ID):

*Protein Expression ~ DE Trait + Individual ID + Residuals*

### LIV-PM differential splicing rate analysis on bulk RNA-seq data

Differential RNA splicing rates were tested between LIV samples and PM samples by adapting the procedures used for differential expression of RNA transcript expression levels described above. The starting point for this analysis was the transcript-level counts matrix for primary RNA and mature RNA output by salmon prior to normalization. Only RNA features present in both the primary RNA and mature RNA expression matrices were included (20,671 RNA transcripts).

In order to normalize the primary RNA and mature RNA matrices together, after subsetting the primary RNA and mature RNA matrices for these features, the two matrices were combined in two different ways to construct a “long” form and a “wide” form. The long form was constructed by concatenating the primary RNA matrix (20,671 rows by 518 columns) and the mature RNA matrix (20,671 rows by 518 columns) into a single matrix of 41,342 rows and 518 columns. In this form, there were two rows for each RNA transcript, and the row name for these two rows were differentiated from each other using an indicator of whether the row represented the primary RNA or the mature RNA for of the transcript (e.g., “FEATURE1-Primary”, “FEATURE1-Mature”). The wide form was constructed by joining the primary RNA matrix and the mature RNA matrix by RNA transcript name into a single matrix of 20,671 rows and 1036 columns. In this form, there was only one row for each RNA transcript (e.g., “FEATURE1”) but there were two columns for each sample, and the column names for these two columns differentiated from each other using an indicator of whether the sample was from the primary RNA or the mature RNA data (“SAMPLE1-Primary”, “SAMPLE1-Mature”). Normalization factors for each of the 518 samples were calculated on the long form of the matrix using the calcNormFactors() functions of the edgeR R package (v3.38.1). The wide form of the matrix was then normalized using these normalization factors with the voomWithDreamWeights() function of the variancePartition R package.

Differential expression was performed on the wide form of the matrix. To do this, a metadata table needed to be constructed that had one row for every column in the wide form of the expression matrix. The columns in the metadata table included LIV-PM status, technical covariates used in differential expression analyses, and a column that indicated whether the row was from the primary RNA or mature RNA data. The formula (“Formula 6” below) for differential splicing rates included an interaction term between the LIV-PM status of the sample and primary-mature RNA status of the sample. Conceptually, the resulting summary statistics from the dream() function of the variancePartition R package represent the differences in the ratio of mature RNA levels (i.e., spliced RNA) to primary RNA levels (i.e., unspliced RNA) between LIV samples and PM samples for each RNA transcript. Positive logFC values mean PM samples have higher splicing rates than LIV samples, and negative logFC values mean that LIV samples have higher splicing rates than PM samples.

Formula 6 (linear mixed model with fixed effects and random effects for individual ID, depletion batch, and sex):

Splicing Rate ~ Spliced Status*LIV-PM Status + Sex + RIN + Neuronal Fraction + Picard RNASeqMetrics Median 3’ Bias + Picard RNASeqMetrics PCT mRNA Bases + Individual ID + Depletion Batch + Picard InsertSizeMetrics Median Insert Size + Picard AlignmentSummaryMetrics Strand Balance (First of Pair) + Residuals

### LIV-PM differential intron usage analysis on bulk RNA-seq data

Leafcutter (v0.2.9) was used to perform differential intron usage analysis on 12,438 intron clusters that remained following filtering procedures described above. LIV samples for this analysis were downsampled to retain one sample per individual due to the inability of the current version of Leafcutter to model the effect of multiple samples per individual (169 LIV samples and 243 PM samples). The sample to retain was chosen randomly for each individual. Differential intron usage was run using the differential_splicing() function of the leafcutter R package (v0.2.9) [13] with the “min_samples_per_intron” and “min_samples_per_group” parameters both set to 10 and “confounders” set to a matrix of covariates that included the same covariates used in Formula 1 above with the exception of Individual ID since there was only one sample per individual used here. Of the 12,438 intron clusters input into the differential intron usage analysis, differential intron usage was successfully run on 11,600 (containing 29,002 of the 32,632 introns) – 808 intron cluster tests failed for timing out and 30 failed for not having enough samples meeting the criteria for testing. After mapping the gene identifiers output by Leafcutter (i.e., gene symbols) to ensembl IDs using the Gencode v30 reference transcriptome source files, 11,222 of the 11,600 intron clusters successfully tested were mapped to an Ensembl gene identifier (6,797 unique Ensembl gene identifiers, meaning some Ensembl gene identifiers contained greater than one intron cluster). The 378 intron clusters without an Ensembl gene identifiers also did not have an associated gene symbol, and these were removed from analysis.

For visualization of differential intron usage in *RSRP1*, the following procedures were followed. The gtf2leafcutter.pl script of the leafcutter package (v0.2.9) was used to generate files for annotating genes in the format required by subsequent leafcutter scripts, using as input the GTF file from the Gencode v30 release. Next, using the resulting annotation files, the intron counts and differential intron usage summary statistics were annotated to genes using the prepare_results.R script of the leafcutter package (v0.2.9), with the “-f” parameter to define significance of intron usage tests set to 0.05. Using the intron counts matrix returned by the prepare_results.R script, for each intron in the *RSRP1* intron cluster the LIV mean count was calculated as the mean of the counts in LIV samples, and the LIV intron usage was calculated as the LIV mean count for the intron divided by the sum of the LIV mean counts for all introns in the cluster. The same procedure was repeated to calculate the PM intron usage for each intron in the cluster. For introns where the LIV mean count was greater than the PM mean count, the intron usage ratio was calculated as the LIV mean count divided by the PM mean count. For introns where the PM mean count was greater than the LIV mean count, the intron usage ratio was calculated as the PM mean count divided by the LIV mean count.

### RNA-protein correlation difference matrices

For the residual mature RNA (30,099) and protein (6,415) expression data, the following procedure was performed to determine if the feature-feature correlations differed between LIV (N = 235 in both RNA and protein) and PM (N = 237 in both RNA and protein) samples:

1. Feature-feature Pearson’s correlation matrices were calculated separately for LIV samples and PM samples using the rcorr() function in the Hmisc (v4.7-0) package in R.
2. The LIV sample feature-feature correlations were subtracted from the PM sample feature-feature correlations and transformed to absolute values, resulting in the “LIV-PM correlation difference matrix.”
3. The mean of the LIV-PM correlation difference matrix was computed.
4. An empirical p-value was calculated to assess the significance of the LIV-PM correlation difference matrix mean. The null distribution used to calculate this p-value was generated through 10,000 permutations of the following five-step procedure: 1) LIV samples were randomly split in half and PM samples were randomly split in half; 2) each of the LIV sample halves was combined with one of the PM sample halves, yielding two “random” sets of samples; 3) feature-feature Pearson’s correlation matrices were calculated for each of the random sample sets using the cor() function in the base stats R package (rcorr was not used here because p-values were not needed); 4) the “null correlation difference matrix” was calculated by subtracting the correlation matrix from one random set from the correlation matrix of the other random set and transforming to absolute values; 5) the null correlation difference matrix mean was computed. The empirical p-value was calculated as the fraction of 10,000 null correlation difference matrix means that were greater than the LIV-PM correlation difference matrix mean.

### LIV-PM differential co-expression analyses on bulk RNA-seq and bulk LC-MS data

A mature RNA-protein Pearson’s correlation matrix (30,099 x 6,415) was calculated separately for LIV (N = 235 with both RNA and protein) and PM (N = 237 with both RNA and protein) samples using the rcorr() function in the Hmisc (v4.7-0) package in R (v4.2.0). A matrix was generated for correlation coefficients and the corresponding p-values. The p-values were adjusted by applying the FDR through the p.adjust() function of the base stats R package (adjusting for 30099 x 6415 = 193,085,085 tests). Adjustment of p-values was performed separately for LIV and PM samples.

RNA-protein pairs were considered “same-gene” pairs if the mature RNA and protein features were derived from the same gene. A total of 5,714 same-gene pairs were present after mapping the 6,415 refseq protein identifiers to ensembl identifiers (successful mapping for 5,758) and subsetting for the ensembl identifiers also present in the 30,099 mature RNA features. The full RNA-protein matrix (30099 x 6415) was subset for these proteins (30099 x 5714 = 171,985,686 RNA-protein correlations).

To test whether same-gene correlations in LIV and PM samples were enriched for being positive and significant, each mature RNA-protein pair was labeled as being 1) a “same-gene” pair and 2) a pair with a statistically significant and positive Pearson’s correlation coefficient (statistical significance here was defined based on the adjusted p-values calculated on the full mature RNA-protein matrix prior to subsetting proteins for the 5714); then, a one-sided Fisher’s exact test was performed to test whether same-gene pairs were enriched for positive-significant pairs using the fisher.test() function in the based stats R package.

The concordance of the same-gene mature RNA-protein correlations in LIV and PM samples was calculated using the cor() function of the base stats R package (Pearson’s). To determine if the LIV-PM status was driving same-gene mature RNA-protein correlations, t-statistics and associated p-values were obtained using the following linear model run using the lm() function of the base stats R package:

Formula 7 (linear model)

*Same-gene correlation coefficient ~ Mature RNA LIV-PM logFC + Protein LIV-PM logFC*

The resulting p-values (4 p-values total, two from the LIV model and 2 from the PM model, one for each logFC variable) were adjusted using the Bonferroni method in the p.adjust() function in the base stats R package. For plotting these results, the mature RNA logFC and the protein logFC were summed, and if the sum was greater than 0 the point was colored blue and if the sum was less than 0 the point was colored pink, then the shade of the point was set to reflect the absolute value of the sum.

To assess whether mature RNA-protein pair correlations differed between LIV (N = 235 in both RNA and protein) and PM (N = 237 in both RNA and protein) samples:

1. The LIV sample mature RNA-protein correlations were subtracted from the PM sample mature RNA-protein correlations and transformed to absolute values, resulting in the “LIV-PM mature RNA-protein correlation difference matrix.”
2. An empirical p-value was calculated for each mature RNA-protein pair in the LIV-PM mature RNA-protein correlation difference matrix to assess the significance of the correlation difference. The null distribution used to calculate this p-value was generated through 10,000 permutations of the following four-step procedure: 1) LIV samples were randomly split in half and PM samples were randomly split in half; 2) each of the LIV sample halves was combined with one of the PM sample halves, yielding two “random” sets of samples; 3) mature RNA-protein Pearson’s correlation for the mature RNA-protein pair of interest was calculated for each of the random sample sets using the cor() function in the base stats R package (R version v4.2.0); 4) the “null correlation difference” was calculated by subtracting the correlation from one random set from the correlation of the other random set and transforming the difference to an absolute value. The empirical p-value was calculated as the fraction of 10,000 null correlation differences that were greater than the LIV-PM correlation difference for the pair of interest.

The percentage of mature RNA-protein pairs that were significantly correlated in either LIV samples or PM samples that were also significantly differentially correlated between LIV samples and PM samples was calculated using the adjusted p-values to define pairs significantly correlated in either LIV or PM (adjusted p-values < 0.05) and using the empirical p-values to define the pairs where the LIV correlation was significantly different than the PM correlation (empirical p-value < 0.05). The OR to test whether the mature RNA-protein pairs that were significantly correlated in either LIV samples or PM samples were enriched for being significantly differentially correlated between LIV samples and PM samples was calculated using the fisher.test() function in the base stats R package as follows:


OR = (a/b)/ (c/d)

*where,*

*a = Number of pairs significantly correlated in LIV or PM and significantly differentially correlated between LIV and PM (N = 1608593).*

*b = Number of pairs significantly correlated in LIV or PM and not significantly differentially correlated between LIV and PM (N = 600205).*

*c = Number of pairs not significantly correlated in LIV or PM and significantly differentially correlated between LIV and PM (N = 14783192).*

*d = Number of pairs not significantly correlated in LIV or PM and not significantly differentially correlated between LIV and PM (N = 176093095).*

To test if mature RNA-protein correlation differences between LIV and PM samples were enriched for protein families defined in KEGG the following approach was taken. KEGG sets labeled as “Protein Families” in the SuperPathwayString field of the KEGG source files were considered for testing, and after filtering for sets that contained at least 10 proteins in the LBP data 36 sets remained for testing. To create the background for these tests a table was created with one row for each of the 2,208,798 mature RNA-protein pairs that were significantly correlated in either LIV or PM samples, with one column for the RNA feature in the pair and one column for the protein feature in the pair. A column was created indicating whether the mature RNA-protein pair was significantly differently correlated between LIV samples and PM samples. For each of the 36 KEGG sets, a column was created indicating whether the protein feature in the pair was a member of the KEGG set. Finally, a Fisher’s exact test was performed to test whether the rows with a TRUE value in the column for differential correlations were enriched for the rows with TRUE values in the KEGG column. Multiple testing correction was done for the 36 tests using the FDR method in the p.adjust() function in the base stats R package.

## Supporting information

S1 File Analyses demonstrating that molecular differences between living and postmortem human brain tissue are not explained by potential confounders. (DOCX)

S1 Fig Barplots showing the number of LIV samples and PM samples (y-axis) in each experimental batch of bulk LC-MS data (x-axis). The top plot shows counts for the full set of PFC samples in the protein LIV-PM DE analysis. The middle plot shows counts for the set of PFC samples in the “batch-matched” protein LIV-PM DE analysis. The bottom plot shows counts for the set of PFC samples in the “batch-unmatched” protein LIV-PM DE analysis. See S1 File for descriptions of how the batch-matched and batch-unmatched sets of samples were defined.
(TIFF)

S2 Fig Density plot showing the distribution of missingness rates for the 10,515 proteins quantified in the bulk LC-MS data prior to quality control. The colors indicate whether the proteins in the distribution were retained for analysis after quality control (red) or were removed from analysis during quality control procedures (gray).
(TIFF)

S3 Fig Example of visualizations used in molecular data quality control (QC) process. For both the RNA sequencing and LC-MS datasets, an iterative procedure was employed to identify technical, biological, and clinical variables (“covariates”) that explain the variance in molecular feature expression between samples that is not due to a variable of interest (in this case, LIV-PM status). The QC process begins by compiling covariates and correlating them to (1) the first five principal components of the molecular expression data, (2) LIV-PM status, and (3) each other. A bioinformatician trained in this QC process then performs a manual review of each covariate’s correlations and selects one covariate to add to the regression model. The molecular data is then transformed by regressing out the effects of the covariates in the updated model, covariate-PC correlations are recalculated using the transformed molecular data, the manual review is performed, and the next covariate is selected. This entire procedure is repeated until the bioinformatician determines that no additional covariates are having a meaningful effect on the variance in molecular expression. The key step of the manual review performed by the bioinformatician involves inspecting a figure such as the one shown here for the covariate “PCT_UTR_bases.” This covariate is calculated using the RnaSeqMetrics tool in the GATK package (indicated by the “RNASeqMetrics_” string in the label) and captures technical aspects of the library preparation step in RNA sequencing. In each plot, a point represents a sample, and the same samples are shown in each of the four plots. Points are colored from green (lower PCT_UTR_bases values) to red (higher PCT_UTR_bases values). Two of the top five principal components of the gene expression data are shown in each plot (one on the x-axis and one on the y-axis). The bioinformatician selected this covariate for inclusion in the regression model primarily due to its visually evident correlation with PC1 in the top left plot (i.e., points with x-axis values above zero are greener and points with x-axis values below zero are redder). The visual analysis of the top right plot also factored into the selection, as it suggested a meaningful correlation between the covariate and PC3.
(PDF)

S4 Fig To ensure the main LIV-PM DE signatures are not explained by differences in the ages of living participants and postmortem donors, LIV-PM DE was performed between: (1) LIV samples with age less than 65 years (N = 151 for RNA and 133 for protein) and PM samples with age less than 65 years (N = 40 for RNA and 37 for proteins; “LIV:*lowAge*-PM:*lowAge* DE”); (2) LIV samples with age greater than or equal to 65 years (N = 124 for RNA and 115 for protein) and PM samples with age greater than or equal to 65 years (N = 203 for RNA and proteins; “LIV:*highAge*-PM:*highAge* DE”). The concordance is plotted between the LIV:*lowAge*-PM:*lowAge* DE signature (x-axis) and the LIV:*highAge*-PM:*highAge* DE signature (y-axis) for Primary RNA, Mature RNA and Protein LIV-PM DE signatures. Each point is a RNA transcript or protein.
(TIFF)

S1 Table The LIV-PM DE signatures from the bulk RNA-seq and bulk LC-MS. (XLSX)

S2 Table KEGG gene set enrichment results for LIV DEFs and PM DEFs in the LIV-PM DE signatures. (XLSX)

S3 Table KEGG enrichments in differentially correlated RNA-protein pairs. (XLSX)
