# Supplementary material for: A study of RNA splicing and protein expression in the living human brain
Source: PLoS One. 2025 Oct 9;20(10):e0332651. doi: 10.1371/journal.pone.0332651 (PMC12510584; doi:10.1371/journal.pone.0332651)
Supplement: S1 File — (DOCX) [file pone.0332651.s001.docx]

**Supplementary Information**

To establish that the LIV-PM DE signatures identified in the main text are not explained by variables with the potential to confound measures of RNA transcript or protein expression, analyses were performed for 12 potential confounding variables:

1. Sample processing batch
2. Institution of origin of the PM samples
3. Postmortem interval (PMI)
4. Diagnosis of PD in living participants and postmortem donors
5. Severity of PD symptoms in living participants
6. Dose of dopamine replacement therapy in living participants
7. Neuropathology in LIV and PM samples
8. Type and dose of anesthesia administered to living participants during DBS surgery
9. Method of LIV sample preservation upon collection during DBS surgery
10. RNA integrity number (RIN)
11. Age differences between living participants and postmortem donors
12. Cell type composition differences between LIV samples and PM samples.

The Supplementary Information presents these analyses split into two sections. The 11 analyses performed in “Concordance of LIV-PM DE signatures: Part 1” used the same analytic framework, whereas the analysis performed in “Concordance of LIV-PM DE signatures: Part 2” used a slightly different analytic framework.

### Concordance of LIV-PM DE signatures: Part 1

This section presents 11 out of the 12 analyses conducted to establish that the primary RNA transcript, mature RNA transcript, and protein LIV-PM DE signatures are not explained by confounding influences on RNA transcript and protein expression. For each of 10 potential confounding variables, a three-step analysis procedure was performed separately for the primary RNA transcript, mature RNA transcript, and protein LIV-PM DE signatures: (1) the LBP cohort was stratified with respect to the potential confounding variable, (2) LIV-PM DE signatures were identified in the resulting groups, (3) the concordance of the LIV-PM DE signatures was assessed with respect to one another or to the “main LIV-PM DE signature” (i.e., the LIV-PM DE signature presented in ***Figure 3***).

1. Sample processing batch

The 518 samples analyzed in the bulk RNA-seq LIV-PM DE analyses will be referred to as “the full LBP transcriptomics cohort.” Bulk RNA-seq of the full LBP transcriptomics cohort was conducted in two waves separated by over one year: the “discovery wave” and “replication wave.” The full LBP transcriptomics cohort was stratified into a discovery wave cohort (49 LIV samples and 57 PM samples) and a replication wave cohort (228 LIV samples and 187 PM samples not in the discovery wave cohort). Primary RNA LIV-PM DE and Mature RNA LIV-PM DE was performed separately for the discovery wave cohort and the replication wave cohort. The concordance between the resulting LIV-PM DE signatures (Primary RNA LIV-PM DE: Spearman’s ρ = 0.86; Mature RNA LIV-PM DE: Spearman’s ρ = 0.78; p-values < 2.2 x 10^-16^) suggests that the Primary RNA and Mature RNA LIV-PM DE signatures are not explained by RNA sequencing wave.

The 488 samples in the bulk LC-MS data analyses will be referred to as “the full LBP proteomics cohort.” Processing of these 488 samples for LC-MS was done in 34 batches (14-15 samples per batch). For technical reasons, it was not possible to for each batch to be comprised of equal numbers of LIV samples and PM samples (***Supplementary Figure 1***). From the full LBP proteomics cohort, a “batch-matched LBP proteomics cohort” and a “batch-unmatched LBP proteomics cohort” were created by applying the following procedure to each batch:

1. The number of LIV samples, N_LIV_, was calculated
2. The number of PM samples, N_PM_, was calculated
3. If N_LIV_ > 0 and N_PM_ = 0, then the LIV samples in the batch were added to the batch-unmatched LBP proteomics cohort
4. If N_LIV_ = 0 and N_PM_ > 0, then the PM samples in the batch were added to the batch-unmatched LBP proteomics cohort
5. If N_LIV_ > 0, N_PM_ > 0, and N_LIV_ < N_PM_, then (a) all LIV samples in the batch were added to the batch-matched LBP proteomics cohort; (b) a randomly selected set of N_LIV_ PM samples were added to the batch-matched LBP proteomics cohort; (c) the PM samples in the batch not added to the batch-matched LBP proteomics cohort were added to the batch-unmatched LBP proteomics cohort
6. If N_LIV_ > 0, N_PM_ > 0, and N_LIV_ > N_PM_, then (a) all PM samples in the batch were added to the batch-matched LBP proteomics cohort; (b) a randomly selected set of N_PM_ LIV samples were added to the batch-matched LBP proteomics cohort; (c) the LIV samples in the batch not added to the batch-matched LBP proteomics cohort were added to the batch-unmatched LBP proteomics cohort.

The resulting batch-matched LBP proteomics cohort had 238 samples (119 LIV samples and 119 PM samples) and the resulting batch-unmatched LBP proteomics cohort had 250 samples (129 LIV samples and 121 PM samples) (***Supplementary Figure 1***). Protein LIV-PM DE was performed separately for the batch-matched LBP proteomics cohort and the batch-unmatched LBP proteomics cohort. The concordance between the resulting LIV-PM DE signatures (Spearman’s ρ = 0.94; p-value < 2.2 x 10^-16^) suggests that the Protein LIV-PM DE signature is not explained by LC-MS sample processing batch.

1. Institution of origin of the PM samples

Institution of origin is a known potential confounder of postmortem brain RNA transcript and protein expression^1^, and PM samples originated from three institutions (i.e., “brain banks”). For this study, frozen postmortem PFC samples were obtained from three separate brain banks: Harvard Brain and Tissue Resource Center (“PM1”; N = 104), the New York Brain Bank at Columbia University (“PM2”; N = 129), and the University of Miami Brain Endowment Bank (“PM3”; N = 13). For primary RNA transcripts, mature RNA transcripts, and proteins, LIV-PM DE was performed between PM1 samples and a random half of the LIV samples and between PM2 samples and the other half of the LIV samples (PM3 samples were not included in this analysis due to small sample size). The concordance between the resulting Primary RNA LIV-PM DE signatures (Spearman’s ρ = 0.73; p-value < 2.2 x 10^-16^), Mature RNA LIV-PM DE signatures (Spearman’s ρ = 0.87; p-value < 2.2 x 10^-16^), and Protein LIV-PM DE signatures (Spearman’s ρ = 0.91; p-value < 2.2 x 10^-16^) suggests that the LIV-PM DE signatures are not explained by brain bank.

1. Postmortem interval (PMI)

Postmortem interval (PMI; i.e., the number of hours elapsed between death and postmortem tissue processing) is another known potential confounder of postmortem RNA transcript and protein expression^2^. Since LIV samples, by definition, do not have a PMI, PMI was not included as a covariate in the LIV-PM DE regression models for each RNA transcript or protein. For both the full LBP transcriptomics cohort and the full LBP proteomics cohort, PM1 samples and PM2 samples were stratified into “low PMI” and “high PMI” subsets, which were defined as the top and bottom quartiles of the PMI distributions, respectively, in each cohort. The resulting PM sample subsets had the following mean PMI values and sample sizes:

| Cohort | Brain Bank | PMI Subset | Mean PMI | Sample Size |
| --- | --- | --- | --- | --- |
| Full LBP transcriptomics cohort | PM1 | Low PMI | 13.11 | 26 |
| Full LBP transcriptomics cohort | PM1 | High PMI | 27.23 | 26 |
| Full LBP transcriptomics cohort | PM2 | Low PMI | 2.15 | 28 |
| Full LBP transcriptomics cohort | PM2 | High PMI | 14.33 | 27 |
| Full LBP proteomics cohort | PM1 | Low PMI | 13.11 | 26 |
| Full LBP proteomics cohort | PM1 | High PMI | 27.36 | 25 |
| Full LBP proteomics cohort | PM2 | Low PMI | 0.94 | 31 |
| Full LBP proteomics cohort | PM2 | High PMI | 13.46 | 31 |

The LIV samples for these analyses were randomly split into four equally or nearly equally sized subsets (for the full LBP transcriptomics cohort: three subsets of 69 samples and one subset of 68; for the full LBP proteomics cohort: four subsets of 62 samples). For primary RNA transcripts, mature RNA transcripts, and proteins, LIV-PM DE was performed between (1) a LIV sample subset and the low PMI subset of PM1 (“LIV-PM:*LowPMI1* DE”), (2) a LIV sample subset and the low PMI subset of PM2 ( “LIV-PM:*LowPMI2* DE”), (3) a LIV sample subset and the high PMI subset of PM1 (“LIV-PM:*HighPMI1* DE”), and (4) a LIV sample subset and the high PMI subset of PM2 (“LIV-PM:*HighPMI2* DE”). The concordance between (a) the LIV-PM:*LowPMI1* DE and LIV-PM:*HighPMI1* DE signatures (Spearman’s ρ = 0.79, 0.76, and 0.80 for Primary RNA, Mature RNA, and Protein LIV-PM DE, respectively; p-values < 2.2 x 10^-16^), and (b) the LIV-PM:*LowPMI2* DE and LIV-PM:*HighPMI2* DE signatures (Spearman’s ρ = 0.85, 0.76, 0.63 for Primary RNA, Mature RNA, and Protein LIV-PM DE, respectively; p-values < 2.2 x 10^-16^) suggests that LIV-PM DE signatures are not simply a recapitulation of the known effect of PMI on postmortem brain RNA transcript and protein expression. This observation is consistent with a previous observation made by the Genotype-Tissue Expression (GTEx) Project using RNA sequencing data from living and postmortem blood samples^3^.

1. Diagnosis of PD in living participants and postmortem donors

The percentage of LIV samples that were obtained from living participants diagnosed with PD (full LBP transcriptomics cohort: 80.00% [220/275]; full LBP proteomics cohort: 79.84% [198/248]) was higher than the percentage of PM samples that were obtained from postmortem donors diagnosed with PD (full LBP transcriptomics cohort: 54.32% [132/243]; full LBP proteomics cohort: 56.25% [135/240]). The full LBP transcriptomics cohort was stratified into a PD cohort (220 “LIV PD samples” and 132 “PM PD samples”) and a non-PD cohort (55 “LIV non-PD samples” and 111 “PM non-PD samples”). Similarly, the full LBP proteomics cohort was stratified into a PD cohort (198 “LIV PD samples” and 135 “PM PD samples”) and a non-PD cohort (50 “LIV non-PD samples” and 105 “PM non-PD samples”). For primary RNA transcripts, mature RNA transcripts, and proteins, LIV-PM DE was performed separately for the PD cohort and the non-PD cohort. The concordance between the resulting Primary RNA LIV-PM DE signatures (Spearman’s ρ = 0.83; p-value < 2.2 x 10^-16^), Mature RNA LIV-PM DE signatures (Spearman’s ρ = 0.79; p-value < 2.2 x 10^-16^), and Protein LIV-PM DE signatures (Spearman’s ρ = 0.70; p-value < 2.2 x 10^-16^) suggests that the LIV-PM DE signatures are not explained by the different percentages of living participants and postmortem donors diagnosed with PD.

1. Severity of PD symptoms in living participants

LIV PD samples were obtained from individuals with varying levels of PD symptom severity while PM PD samples were obtained from individuals whose PD symptom severity at the time of death was not provided by the brain banks. Living individuals with PD were categorized by PD symptom severity using scores from the Unified Parkinson’s Disease Rating Scale Motor subscale (UPDRS-III). Symptom severity was measured when individuals were off dopamine replacement therapy prior to the first DBS surgery (median time between symptom severity measurement and first DBS surgery = 60 days). UPDRS-III scores less than 40 were defined as low and UPDRS-III scores greater than or equal to 40 were defined as high based on manual inspection of the distribution of the UPDRS-III scores.

For primary RNA transcripts, mature RNA transcripts, and proteins, LIV-PM DE was performed between (1) LIV samples from individuals with low UPDRS-III scores (N = 92 samples from 60 individuals for RNA; N = 88 samples from 57 individuals for protein) and a random half of the PM PD samples (N = 66 for RNA; N = 67 for protein; “LIV:*lowUPDRS*-PM DE”), and (2) LIV samples from individuals with high UPDRS-III scores (N = 83 samples from 47 individuals for RNA; N = 71 samples from 43 individuals for protein) and the other half of the PM PD samples (N = 66 for RNA; N = 68 for protein; “LIV:*highUPDRS*-PM DE”). The concordance between the main LIV-PM DE signature and (1) the LIV:*lowUPDRS*-PM DE signature (Spearman’s ρ = 0.93, 0.91, and 0.94 for Primary RNA, Mature RNA, and Protein LIV-PM DE, respectively; p-values < 2.2 x 10^-16^), and (2) the LIV:*highUPDRS*-PM DE signature (Spearman’s ρ = 0.95, 0.91, and 0.77 for Primary RNA, Mature RNA, and Protein LIV-PM DE, respectively; p-values < 2.2 x 10^-16^) suggests that LIV-PM DE signatures are not explained by PD symptom severity in the living cohort.

1. Dose of dopamine replacement therapy in living participants

LIV PD samples were obtained from individuals chronically receiving dopamine replacement therapy (i.e., levodopa) while PM PD samples were obtained from individuals whose dopamine replacement therapy status at the time of death was not provided by the brain banks. LIV PD samples in this analysis were limited to the samples with available levodopa dosing information (for RNA sequencing: 216 LIV PD samples from 130 individuals; for LC-MS: 206 LIV PD samples from 122 individuals). Levodopa dose (i.e., the dose of levodopa taken by a living participant at a time point close to LIV PD sample collection) less than 900 mg was defined as low and levodopa dose greater than or equal to 900 mg was defined as high based on the median of the distribution of the levodopa doses. For primary RNA transcripts, mature RNA transcripts, and proteins, LIV-PM DE was performed between (1) LIV PD samples with low levodopa dose (N = 104 for RNA; N = 100 for protein) and a random half of the PM samples (N = 121 for RNA; N = 120 for protein; “LIV:*lowDOPA*-PM DE”), and (2) LIV PD samples with high levodopa dose (N = 112 for RNA; N = 106 for protein) and the other half of the PM samples (N = 122 for RNA; N = 120 for protein; “LIV:*highDOPA*-PM DE”). The concordance between the main LIV-PM DE signature and (1) the LIV:*lowDOPA*-PM DE signature (Spearman’s ρ = 0.98, 0.97, and 0.95 for Primary RNA, Mature RNA, and Protein LIV-PM DE, respectively; p-values < 2.2 x 10^-16^), and (2) the LIV:*highDOPA*-PM DE signature (Spearman’s ρ = 0.98, 0.97, and 0.97 for Primary RNA, Mature RNA, and Protein LIV-PM DE, respectively; p-values < 2.2 x 10^-16^) suggests that LIV-PM DE signatures are not explained by levodopa dose.

1. Neuropathology in LIV and PM samples

The living and postmortem cohorts included individuals with various degrees of neuropathology as measured on whole slide images by five immunohistochemistry stains (decribed in the methods section). A sample was defined as “neuropathology-free” if no evidence of neuropathology was found on any of the five stains. For bulk RNA-seq, this resulted in 19 LIV samples and 7 PM samples retained for analysis. For bulk LC-MS, this resulted in 18 LIV samples and 7 PM samples retained for analysis. LIV-PM DE was performed on the neuropathology-free subset of samples, resulting in a “neuropathology-free LIV-PM DE signature” for primary RNA transcripts, mature RNA transcripts, and proteins. The concordance (Spearman’s ρ) between the main LIV-PM DE signature and the neuropathology-free LIV-PM DE signature was 0.78 for primary RNA transcripts, 0.60 for mature RNA transcripts, and 0.79 for proteins (all p-values < 2.2 x 10^-16^). These findings suggest that the main LIV-PM DE signatures are not explained by the degree of neuropathology present in LIV and PM samples.

1. Type and dose of anesthesia administered to living participants during DBS surgery

LIV samples were obtained from individuals under anesthesia during DBS surgery while PM samples were obtained from individuals whose anesthesia status at the time of death was not provided by the brain banks. Up to three types of anesthesia were given to each living individual: propofol, fentanyl, and dexmedetomidine. For the three types of anesthesia given, the following four-step procedure was performed separately for primary RNA transcripts, mature RNA transcripts, and proteins:

1. LIV samples with available anesthesia data (N = 281) were split into quartiles representing four dose ranges of the anesthetic (N = 70 or N = 71 LIV samples in each quartile), then each quartile was subset for the samples in the full LBP cohort
2. PM samples in the full LBP cohort were randomly split into four groups (N = 60 or N = 61 PM samples in each group)
3. LIV-PM DE was performed between each quartile and one of the four PM groups.

Performing this procedure produced 12 LIV-PM DE signatures for each of the three types of anesthesia (i.e., for each of the four quartiles, one Primary RNA LIV-PM DE signature, one Mature RNA LIV-PM DE signature, and one Protein LIV-PM DE signature), for a total of 36 LIV-PM DE signatures (e.g., for Primary RNA – LIV*:PropofolQ1*-PM DE, LIV:*PropofolQ2*-PM DE, LIV:*PropofolQ3*-PM DE, LIV:*PropofolQ4*-PM DE, LIV*:DexmedetomidineQ1*-PM DE, LIV*:DexmedetomidineQ2*-PM DE, LIV*:DexmedetomidineQ3*-PM DE, LIV*:DexmedetomidineQ4*-PM DE, LIV*:FentanylQ1*-PM DE, LIV*:FentanylQ2*-PM DE, LIV:*FentanylQ3*-PM DE, LIV:*FentanylQ4*-PM DE). The concordance between these 36 LIV-PM DE signatures and the main Primary RNA, Mature RNA, and Protein LIV-PM DE signatures (Primary RNA: Spearman’s ρ range = 0.91 – 0.97; Mature RNA: Spearman’s ρ range = 0.95 – 0.97; Protein: Spearman’s ρ range = 0.80 – 0.94; all p-values < 2.2 x 10^-16^) suggests that the main LIV-PM DE signatures are not explained by the type and dose of anesthesia given to living individuals.

1. Method of LIV sample preservation upon collection during DBS surgery

The method of preservation used for each LIV sample obtained during DBS surgery was either (1) placement in a tube of RNAlater or (2) placement in a tube on dry ice. DE was performed between (1) the LIV samples preserved by placement in RNAlater (N = 189 for RNA and 167 for protein) and a random half of the PM samples (N = 122 for RNA and 120 for protein; “LIV:*RNAlater*-PM DE”), and (2) the LIV samples preserved by placement on dry ice (N = 86 for RNA and 81 for protein) and the other half of the PM samples (N = 121 for RNA and 120 for protein; “LIV:*DryIce*-PM DE”). The concordance between the main LIV-PM DE signatures and (1) the LIV:*RNAlater*-PM DE signatures (Spearman’s ρ = 0.98, 0.97, 0.93 for Primary RNA, Mature RNA and Protein LIV-PM DE signatures, respectively; all p-values < 2.2 x 10^-16^), and (2) the LIV:*DryIce*-PM DE signature (Spearman’s ρ = 0.94, 0.90, 0.81 for Primary RNA, Mature RNA and Protein LIV-PM DE signatures, respectively; all p-values < 2.2 x 10^-16^) suggests that the main LIV-PM DE signatures are not explained by the method of LIV sample preservation.

1. RNA integrity number (RIN)

RIN, a known confounder of RNA sequencing data and a measure of tissue quality, was included as a covariate in the model used to discover the main Primary and Mature RNA LIV-PM DE signatures. This was done so that associations identified between LIV-PM status and gene expression levels are independent of associations between RIN and gene expression levels. RIN of LIV samples ranged from 5.5 to 8.8 and RIN of PM samples ranged from 4.1 to 9.7. For Primary RNA, Mature RNA, and Protein LIV-PM DE additionally was performed between (1) the LIV samples with RIN less than or equal to 7 (N = 117 for RNA and 94 for protein) and PM samples with RIN less than or equal to 7 (N = 69 for RNA and 68 for protein; “LIV:*lowRIN*-PM:*lowRIN* DE”), and (2) the LIV samples with RIN greater than 7 (N = 158 for RNA and 154 for protein) and PM samples with RIN greater than 7 (N = 174 for RNA and 172 for protein; “LIV:*highRIN*-PM:*highRIN* DE”). The concordance between the main LIV-PM DE signatures and (1) the LIV:*lowRIN*-PM:*lowRIN* DE signature (Spearman’s ρ = 0.95, 0.94, 0.94 for Primary RNA, Mature RNA and Protein LIV-PM DE signatures, respectively; all p-values < 2.2 x 10^-16^), and (2) the LIV:*highRIN*-PM:*highRIN* DE (Spearman’s ρ = 0.98 for the Primary RNA, Mature RNA and Protein LIV-PM DE signatures; all p-values < 2.2 x 10^-16^) suggests that the LIV-PM DE signatures are not explained by RIN.

1. Age differences between living participants and postmortem donors

Age at the time of tissue sampling is often accounted for as a covariate in DE analyses of human brain phenotypes due to the known relationship between age and human brain gene expression^4^. The iterative procedure employed for identification of confounder variables did not identify age as a covariate to be included in the model used to discover the main LIV-PM DE signatures. To ensure the main LIV-PM DE signatures are not explained by differences in the ages of living participants and postmortem donors, LIV-PM DE was performed between:

1. LIV samples with age less than 65 years (N = 151 for RNA and 133 for protein) and PM samples with age less than 65 years (N = 40 for RNA and 37 for proteins; “LIV:*lowAge*-PM:*lowAge* DE”)
2. LIV samples with age greater than or equal to 65 years (N = 124 for RNA and 115 for protein) and PM samples with age greater than or equal to 65 years (N = 203 for RNA and proteins; “LIV:*highAge*-PM:*highAge* DE”)
3. LIV samples with age less than 65 years and PM samples with age greater than or equal to 65 years (“LIV:*lowAge*-PM:*highAge* DE”)
4. LIV samples with age greater than or equal to 65 years and the PM samples with age less than 65 years (“LIV:*highAge*-PM:*lowAge* DE”).

The concordance between (1) the LIV:*lowAge*-PM:*lowAge* DE signature and the LIV:*highAge*-PM:*highAge* DE signature (Spearman’s ρ = 0.86, 0.81, 0.84 for Primary RNA, Mature RNA and Protein LIV-PM DE signatures, respectively; all p-values < 2.2 x 10^-16^), (2) the LIV:*lowAge*-PM:*lowAge* DE signature and the LIV:*highAge*-PM:*lowAge* DE signature (Spearman’s ρ = 0.95, 0.94, 0.95 for Primary RNA, Mature RNA and Protein LIV-PM DE signatures, respectively; all p-values < 2.2 x 10^-16^), (3) the LIV:*lowAge*-PM:*lowAge* DE signature and the LIV:*lowAge*-PM:*highAge* DE signature (Spearman’s ρ = 0.88, 0.85, 0.75 for Primary RNA, Mature RNA and Protein LIV-PM DE signatures, respectively; all p-values < 2.2 x 10^-16^), (4) the LIV:*highAge*-PM:*highAge* DE signature and the LIV:*highAge*-PM:*lowAge* DE signature (Spearman’s ρ = 0.87, 0.84, 0.81 for Primary RNA, Mature RNA and Protein LIV-PM DE signatures, respectively; all p-values < 2.2 x 10^-16^), (5) the LIV:*highAge*-PM:*highAge* DE signature and the LIV:*lowAge*-PM:*highAge* DE signature (Spearman’s ρ = 0.97, 0.95, 0.91 for Primary RNA, Mature RNA and Protein LIV-PM DE signatures, respectively; all p-values < 2.2 x 10^-16^), and (6) the LIV:*highAge*-PM:*lowAge* DE signature and the LIV:*lowAge*-PM:*highAge* DE signature (Spearman’s ρ = 0.83, 0.78, 0.60 for Primary RNA, Mature RNA and Protein LIV-PM DE signatures, respectively; all p-values < 2.2 x 10^-16^) suggests that the LIV-PM DE signature is not explained by age differences between living individuals and postmortem donors.

### Concordance of LIV-PM DE signatures: Part 2

This section of the Supplementary Information presents the final of the 12 analyses conducted to establish that the main LIV-PM DE signatures are not explained by potential confounding influences on RNA transcript and protein expression.

1. Cell type composition differences between LIV samples and PM samples

Cell fractions for the samples in the full LBP cohort were estimated using a transcriptome-based cell type reference^5^ comprised of five cell types: glutamatergic neurons (GLU), GABA-ergic neurons (GABA), oligodendrocytes (ODC), astrocytes (AST), and microglia (MG). Neuronal cell fraction estimates were calculated by summing the GLU and GABA fraction estimates. Quality control procedures described in the methods section identified (1) neuronal cell fraction as a covariate to be included in the model used to discover the main RNA transcript LIV-PM DE signatures and (2) no cell fraction covariates to be included in the model used to discover the main Protein LIV-PM DE signature. Many additional possible combinations of cell fractions could have theoretically been used in these models (i.e., all combinations noted in the table below). To further establish that the main LIV-PM DE signatures are not explained by cell type composition, LIV-PM DE was performed for all possible combinations of cell fractions (for one combination – neuronal, ODC, and AST – it was not possible to perform RNA transcript LIV-PM DE for statistical reasons [i.e., the co-linearity between co-linearity between these variables]). The concordance between the main LIV-PM DE signatures and the resulting LIV-PM DE signatures, presented in the following table, suggests that the main LIV-PM DE signatures are not explained by cell type composition. The table column descriptions are:

- “LIV-PM DE” – the data input for DE analysis for the LIV-PM DE signature reported in the row
- “Cell fractions” – the cell fractions included as covariates in the model used to identify the LIV-PM DE signature reported in the row
- “Concordance” – the Spearman’s correlation coefficient (ρ) between the primary LIV-PM DE signature and the LIV-PM DE signature reported in the row
- “Fraction DEG” – the fraction of RNA transcripts or proteins expressed in the full LBP cohort that were identified as LIV-PM DEGs in the LIV-PM DE signature reported in the row.

| LIV-PM DE | Cell fractions | Concordance | Fraction DEG |
| --- | --- | --- | --- |
| Mature RNA | AST, MG | 0.96 | 0.70 |
| Mature RNA | AST, ODC, MG | 0.99 | 0.70 |
| Mature RNA | AST, ODC | 1.00 | 0.70 |
| Mature RNA | AST | 0.85 | 0.70 |
| Mature RNA | MG | 0.97 | 0.70 |
| Mature RNA | Neuronal, AST, MG | 0.99 | 0.70 |
| Mature RNA | Neuronal, AST | 1.00 | 0.70 |
| Mature RNA | Neuronal, MG | 1.00 | 0.70 |
| Mature RNA | Neuronal, ODC, MG | 0.99 | 0.70 |
| Mature RNA | Neuronal, ODC | 1.00 | 0.70 |
| Mature RNA | ODC, MG | 1.00 | 0.70 |
| Mature RNA | ODC | 1.00 | 0.70 |
| Primary RNA | AST, MG | 0.96 | 0.74 |
| Primary RNA | AST, ODC, MG | 0.99 | 0.74 |
| Primary RNA | AST, ODC | 1.00 | 0.74 |
| Primary RNA | AST | 0.82 | 0.74 |
| Primary RNA | MG | 0.96 | 0.74 |
| Primary RNA | Neuronal, AST, MG | 0.99 | 0.74 |
| Primary RNA | Neuronal, AST | 0.99 | 0.74 |
| Primary RNA | Neuronal, MG | 1.00 | 0.74 |
| Primary RNA | Neuronal, ODC, MG | 0.99 | 0.74 |
| Primary RNA | Neuronal, ODC | 1.00 | 0.74 |
| Primary RNA | ODC, MG | 1.00 | 0.74 |
| Primary RNA | ODC | 1.00 | 0.74 |
| Protein | AST, MG | 0.98 | 0.61 |
| Protein | AST, ODC, MG | 0.97 | 0.61 |
| Protein | AST, ODC | 0.98 | 0.61 |
| Protein | AST | 1.00 | 0.61 |
| Protein | MG | 0.99 | 0.61 |
| Protein | Neuronal, AST, MG | 0.97 | 0.61 |
| Protein | Neuronal, AST, ODC | 0.97 | 0.61 |
| Protein | Neuronal, AST | 0.98 | 0.61 |
| Protein | Neuronal, MG | 0.98 | 0.61 |
| Protein | Neuronal, ODC, MG | 0.97 | 0.61 |
| Protein | Neuronal, ODC | 0.98 | 0.61 |
| Protein | Neuronal | 0.98 | 0.61 |
| Protein | ODC, MG | 0.98 | 0.61 |
| Protein | ODC | 0.98 | 0.61 |

## References

1 Beckmann, N. D. *et al.* Multiscale causal networks identify VGF as a key regulator of Alzheimer's disease. *Nat Commun* **11**, 3942 (2020). <https://doi.org:10.1038/s41467-020-17405-z>

2 Stan, A. D. *et al.* Human postmortem tissue: what quality markers matter? *Brain Res.* **1123**, 1 (2006). <https://doi.org:10.1016/j.brainres.2006.09.025>

3 Ferreira, P. G. *et al.* The effects of death and post-mortem cold ischemia on human tissue transcriptomes. *Nat Commun* **9**, 490 (2018). <https://doi.org:10.1038/s41467-017-02772-x>

4 Ham, S. & Lee, S. V. Advances in transcriptome analysis of human brain aging. *Exp Mol Med* **52**, 1787-1797 (2020). <https://doi.org:10.1038/s12276-020-00522-6>

5 Lake, B. B. *et al.* Integrative single-cell analysis of transcriptional and epigenetic states in the human adult brain. *Nature biotechnology* **36**, 70-80 (2018). <https://doi.org:10.1038/nbt.4038>
