# Supplementary figures and images for: A study of RNA splicing and protein expression in the living human brain
Source: PLoS One. 2025 Oct 9;20(10):e0332651. doi: 10.1371/journal.pone.0332651 (PMC12510584; doi:10.1371/journal.pone.0332651)

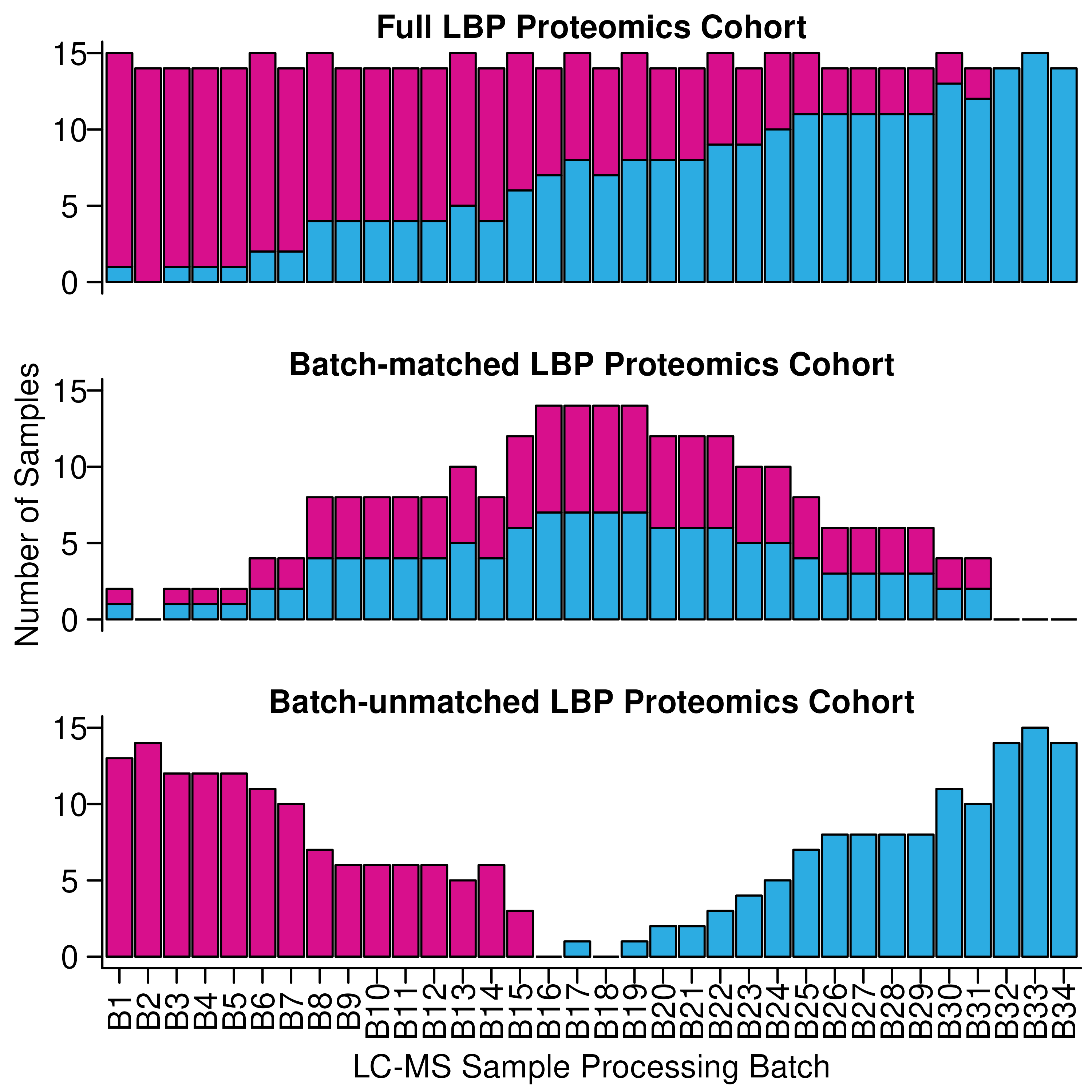

Supplement: S1 Fig — The top plot shows counts for the full set of PFC samples in the protein LIV-PM DE analysis. The middle plot shows counts for the set of PFC samples in the “batch-matched” protein LIV-PM DE analysis. The bottom plot shows counts for the set of PFC samples in the “batch-unmatched” protein LIV-PM DE analysis. See S1 File for descriptions of how the batch-matched and batch-unmatched sets of samples were defined. (TIFF) [file pone.0332651.s002.tiff]

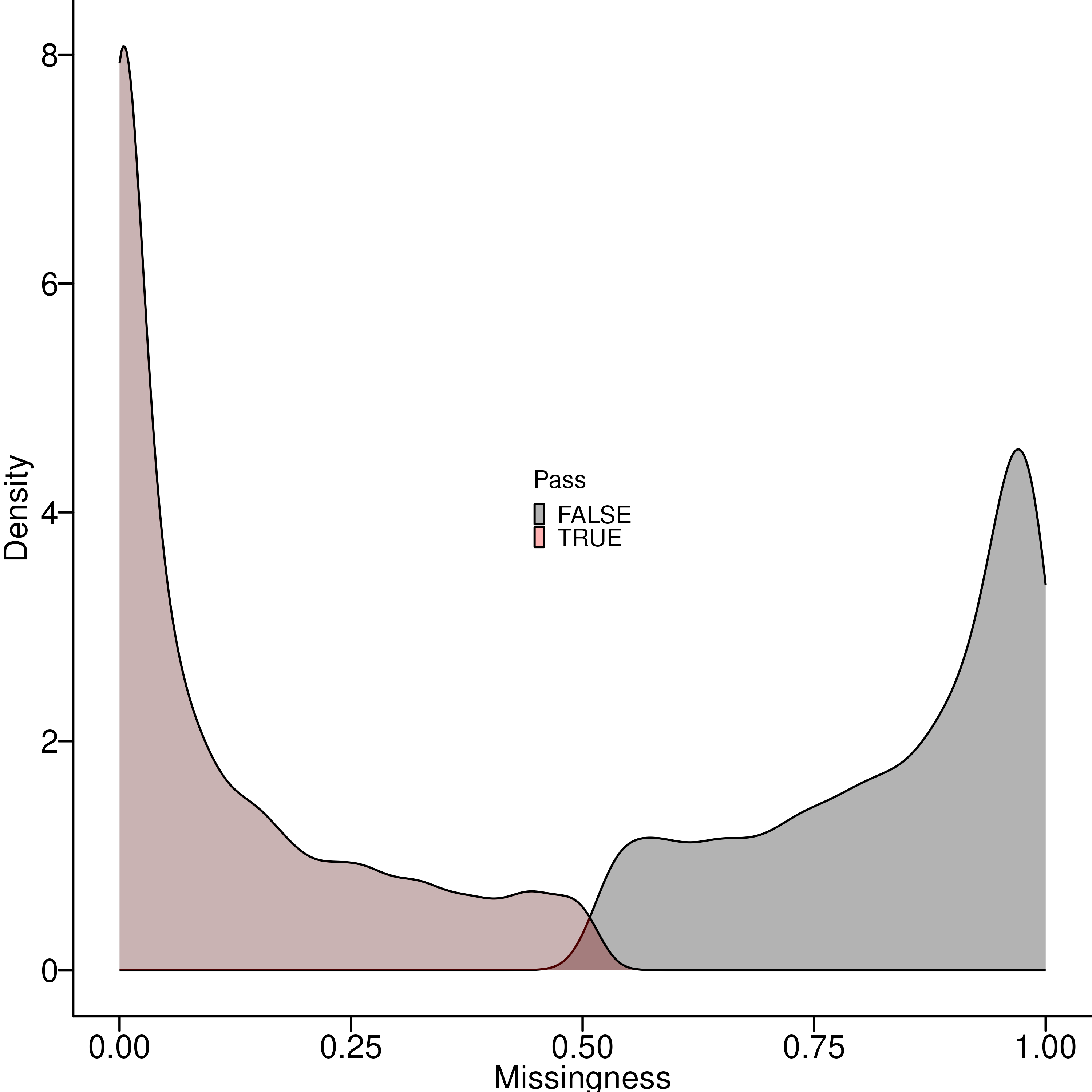

Supplement: S2 Fig — The colors indicate whether the proteins in the distribution were retained for analysis after quality control (red) or were removed from analysis during quality control procedures (gray). (TIFF) [file pone.0332651.s003.tiff]

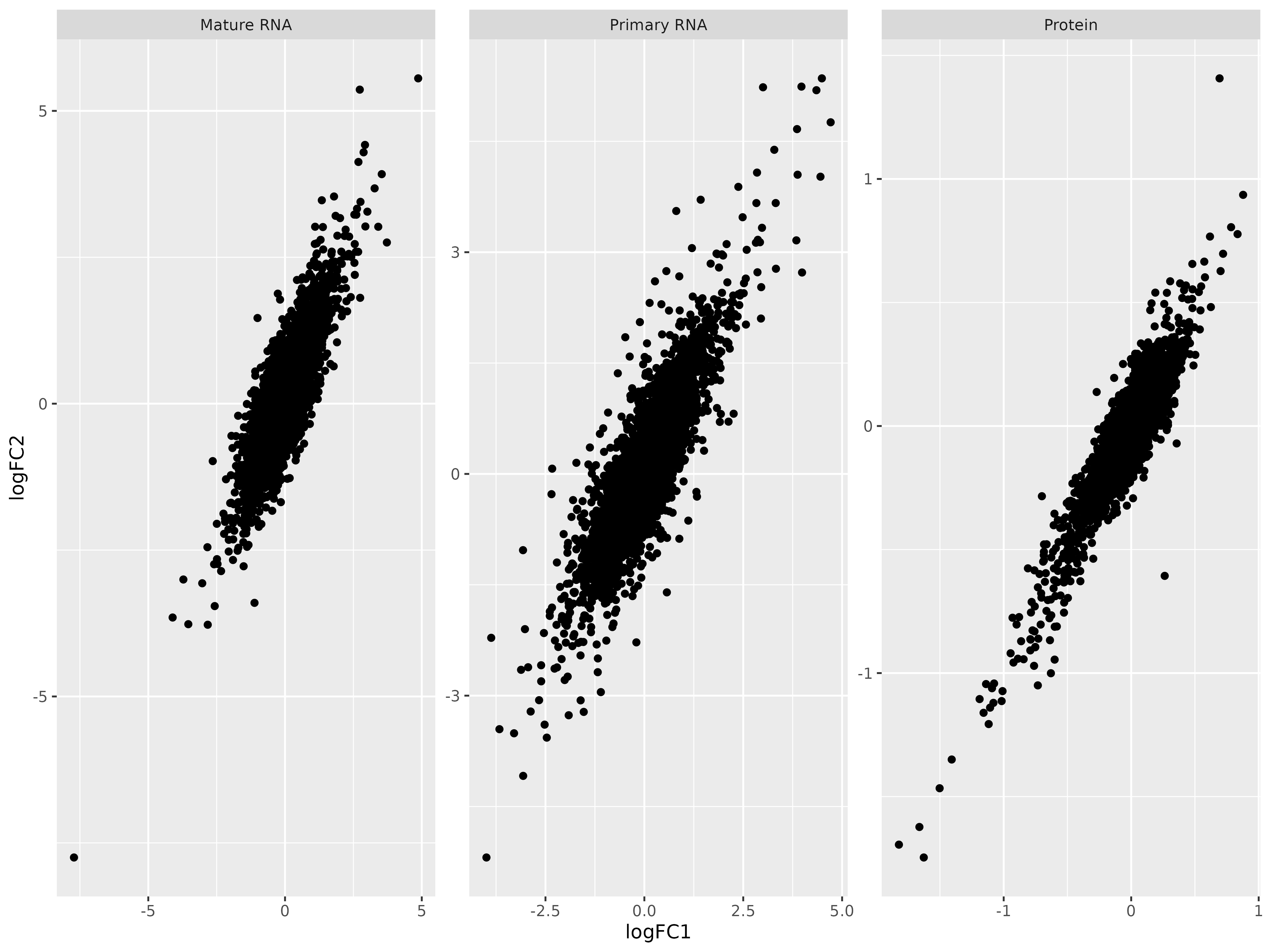

Supplement: S4 Fig — The concordance is plotted between the LIV:lowAge-PM:lowAge DE signature (x-axis) and the LIV:highAge-PM:highAge DE signature (y-axis) for Primary RNA, Mature RNA and Protein LIV-PM DE signatures. Each point is a RNA transcript or protein. (TIFF) [file pone.0332651.s005.tiff]
