# Supplementary material for: A study of RNA splicing and protein expression in the living human brain
Source: PLoS One. 2025 Oct 9;20(10):e0332651. doi: 10.1371/journal.pone.0332651 (PMC12510584; doi:10.1371/journal.pone.0332651)
Supplement: S3 Fig — For both the RNA sequencing and LC-MS datasets, an iterative procedure was employed to identify technical, biological, and clinical variables (“covariates”) that explain the variance in molecular feature expression between samples that is not due to a variable of interest (in this case, LIV-PM status). The QC process begins by compiling covariates and correlating them to (1) the first five principal components of the molecular expression data, (2) LIV-PM status, and (3) each other. A bioinformatician trained in this QC process then performs a manual review of each covariate’s correlations and selects one covariate to add to the regression model. The molecular data is then transformed by regressing out the effects of the covariates in the updated model, covariate-PC correlations are recalculated using the transformed molecular data, the manual review is performed, and the next covariate is selected. This entire procedure is repeated until the bioinformatician determines that no additional covariates are having a meaningful effect on the variance in molecular expression. The key step of the manual review performed by the bioinformatician involves inspecting a figure such as the one shown here for the covariate “PCT_UTR_bases.” This covariate is calculated using the RnaSeqMetrics tool in the GATK package (indicated by the “RNASeqMetrics_” string in the label) and captures technical aspects of the library preparation step in RNA sequencing. In each plot, a point represents a sample, and the same samples are shown in each of the four plots. Points are colored from green (lower PCT_UTR_bases values) to red (higher PCT_UTR_bases values). Two of the top five principal components of the gene expression data are shown in each plot (one on the x-axis and one on the y-axis). The bioinformatician selected this covariate for inclusion in the regression model primarily due to its visually evident correlation with PC1 in the top left plot (i.e., points with x-axis values above zero [file pone.0332651.s004.pdf]

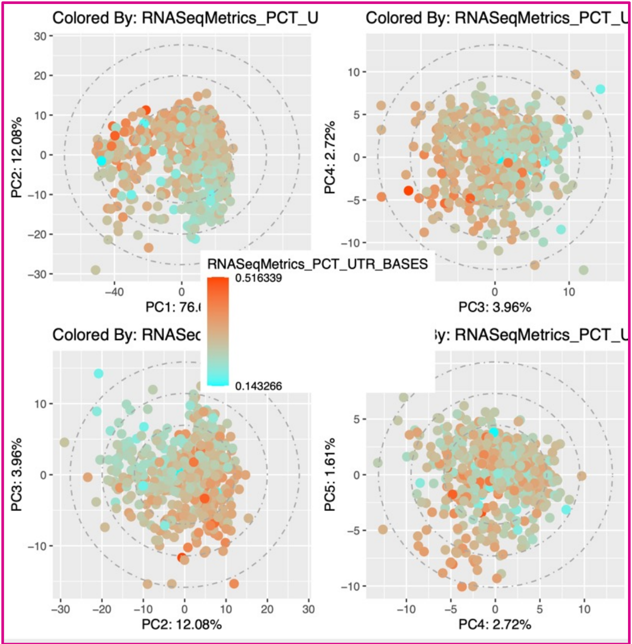

Colored By: RNASeqMetrics_PCT_U
PC2: 12.08%
PC1: 76.(
Colored By: RNASeqMetrics_PCT_U
PC4: 2.72%
PC3: 3.96%
RNASeqMetrics_PCT_UTR_BASES
0.516339
0.143266
Colored By: RNASec
PC3: 3.96%
PC2: 12.08%
3y: RNASeqMetrics_PCT_U
PC5: 1.61%
PC4: 2.72%
